# Methylation-mediated LINC00261 suppresses pancreatic cancer progression by epigenetically inhibiting c-Myc transcription

**DOI:** 10.7150/thno.44278

**Published:** 2020-08-25

**Authors:** Songsong Liu, Yao Zheng, Yujun Zhang, Junfeng Zhang, Fuming Xie, Shixiang Guo, Jianyou Gu, Jiali Yang, Ping Zheng, Jiejuan Lai, Liangyu Yin, Huaizhi Wang

**Affiliations:** 1 Institute of Hepatopancreatobiliary Surgery, Southwest Hospital, Third Military Medical University (Army Medical University), Chongqing, P. R. China; 2 Institute of Hepatopancreatobiliary Surgery, Chongqing General Hospital, University of Chinese Academy of Sciences, Chongqing, P. R. China; 3 Department of First Hepatobiliary Surgery, Zhujiang Hospital, Southern Medical University, Guangzhou, Guangdong, P. R. China; 4 Department of Clinical Nutrition, Daping Hospital, Third Military Medical University (Army Medical University), Chongqing, P. R. China

**Keywords:** LINC00261, c-Myc, Pancreatic cancer, p300/CBP, H3K27Ac

## Abstract

**Background:** Due to the limitations of strategies for its early diagnosis and treatment, pancreatic cancer (PC) remains a substantial human health threat. We previously discovered a methylation-mediated lncRNA, LINC00261, which is downregulated in PC tissues. However, the underlying role of LINC00261 in PC remains largely unknown.

**Methods:** Quantitative real-time PCR and in situ hybridization were performed to evaluate the expression levels of LINC00261 in PC, adjacent nontumor and normal pancreas tissues. The clinical significance of LINC00261 was assessed in multicenter PC samples. The functions of LINC00261 in PC were investigated by gain- and loss-of-function assays *in vitro* and *in vivo*. Potential downstream pathways and mechanisms were explored via RNA sequencing and bioinformatic analyses. RNA immunoprecipitation and chromatin immunoprecipitation assays were used to validate the underlying mechanisms. Pyrosequencing and targeted demethylation of the LINC00261 promoter were performed to explore the upstream epigenetic mechanisms and therapeutic potential.

**Results:** LINC00261 was significantly downregulated in PC tissues, and its expression was positively associated with the prognosis of PC patients. Phenotypic studies indicated that LINC00261 overexpression significantly suppressed PC cell proliferation, migration and metastasis *in vitro* and *in vivo*. c-Myc was identified as a downstream target of LINC00261. LINC00261 repressed c-Myc transcription by physically interacting and binding with the bromo domain of p300/CBP, preventing the recruitment of p300/CBP to the promoter region of c-Myc and decreasing the H3K27Ac level. Moreover, the methylation level of the LINC00261 promoter was high in PC tissues and was correlated with poor prognosis. Targeted demethylation of the LINC00261 promoter inhibited PC progression both *in vitro* and *in vivo*.

**Conclusions:** Our findings indicate that methylation-mediated LINC00261 suppresses PC progression by epigenetically repressing c-Myc expression. These findings expand the therapeutic potential of LINC00261, possibly providing evidence to support the development of epigenetic drugs or therapeutic strategies. This research adds further insights into the etiology of PC and indicates that LINC00261 may be a prognostic and therapeutic target in PC.

## Introduction

According to the latest global cancer statistics, pancreatic cancer (PC) is the seventh leading cause of cancer-related death 1. Due to the limitations of strategies for its early diagnosis and treatment, the five-year survival rate for PC patients is less than 7% 2. Moreover, the incidence and mortality rates of PC continue to increase 3; thus, novel therapeutic targets and derived treatment strategies are urgently needed for physicians and patients.

Recent studies have shown that lncRNA dysregulation is involved in various malignancies, such as lung cancer 4, esophageal cancer 5, gastric cancer 6, liver cancer 7 and PC 8, 9, by affecting diverse tumor biological behaviors, such as proliferation, invasion, migration and metastasis, both* in vitro* and* in vivo.* Increasing numbers of lncRNA studies in PC have been reported 9-12. However, detailed evidence elucidating the underlying functions of lncRNAs, especially their regulatory roles in driving the key proto-oncogenes and oncogenes in PC—such as Kras 11, p53 12 and c-Myc 13—remains scarce.

Epigenetic regulation of lncRNAs has been suggested to be an important mechanism contributing to cancer progression 14. Although lncRNAs can act as tumor suppressors in various cancers 15, some lncRNAs with tumor suppressor capability might be inactivated in cancers due to methylation of their promoter regions 16. Thus, the application of targeted demethylation techniques 17 to these tumor suppressor lncRNAs might contribute to the discovery of novel treatment methods such as epigenetic drugs.

We applied data mining techniques to public Gene Expression Omnibus (GEO) datasets of lncRNA microarray data to identify differentially expressed lncRNAs between PC and normal pancreas (NP) tissues. Among the numerous dysregulated lncRNAs, the DNA-methylated LINC00261 exhibited markedly lower expression levels in PC tissue than in NP tissue. Previous studies showed that LINC00261 might be a biomarker 18 and metastasis inhibitor in PC 19, 20. However, these studies were performed *in vitro*, possibly limiting the power of their results, and the prognostic value of LINC00261 has not been directly validated in multicenter PC cohorts. More importantly, the upstream and downstream mechanisms underlying the epigenetic role and potential therapeutic utility of LINC00261 remain unclear. To address these issues, we aimed to comprehensively investigate the molecular mechanisms related to LINC00261 and the potential therapeutic and prognostic value of this lncRNA in PC.

## Materials and Methods

### Cohorts and tissue samples

All fresh-frozen tissue samples, including the PC tissues with adjacent noncancerous tissues used for qPCR (30 NP tissues and 150 PC tissues, 40 pairs of PC and adjacent nontumor tissues), were obtained from the Institute of Hepatopancreatobiliary Surgery, Southwest Hospital, Army Medical University. Of the 298 formalin-fixed, paraffin-embedded tissues contained in the two independent tissue microarrays, 55 PC (Cohort 1) and 13 NP tissues were obtained from the archival collections of Southwest Hospital, 50 PC tissues were obtained from Soochow University (Cohort 2) and 100 PC and 80 NP tissues were purchased from Outdo Biotech (Shanghai, China) (Cohort 3). For the 850k microarray, 28 PC tissues and 18 adjacent noncancerous tissues were obtained from the Southwest Hospital. NP samples were obtained from organ donors. The pathological type of all PC tissues in the present study was pancreatic ductal adenocarcinoma, and diagnoses were made based on surgical pathology. No patient received either chemotherapy or radiotherapy before surgery. The clinical stage was evaluated based on the guidelines in the American Joint Committee on Cancer (AJCC) 7^th^ edition. Resected specimens were cut into blocks of a proper size, immediately submerged in the RNA preserving reagent RNAlater (Thermo, USA) and either frozen in liquid nitrogen for further RNA extraction and qPCR analysis or formaldehyde fixed and paraffin embedded for further histological analysis. Each sample was used for only one specific type of assay, for example, in situ hybridization (ISH) or qPCR. Follow-up was performed every three months after surgery to obtain the survival status. The study protocol was approved by the Ethics Committee of Southwest Hospital (No. KY201875), Army Medical University. All patients provided written informed consent upon admission for the use of human specimens. All procedures, including the use of human tissue specimens and analysis of clinical data, were carefully handled to meet the guidelines of the Declaration of Helsinki.

### *In vivo* animal experiments

Four- to six-week-old female nude mice were purchased from Southwest Hospital (Chongqing, China). All animal experimental procedures were carried out under aseptic conditions. To establish the subcutaneously implanted tumor model, 2×10^6^ cells (in a total volume of 0.1 ml of PBS) were injected into the dorsal region of each mouse at the sixth week. The body weight and tumor growth of each mouse were measured weekly. All efforts were made to minimize suffering, and all mice were sacrificed for measurement of tumor weights 5 weeks after establishment of the model. To establish the metastasis model in nude mice, a midline incision was made in the anterior abdominal wall, and 2×10^6^ cells (in a total volume of 0.1 ml of PBS) were directly injected into the distal pancreatic parenchyma at the sixth week. Mice were anesthetized with isoflurane inhalation or pentobarbital sodium. After 6 weeks, the liver metastatic ability of the PC cells was observed by harvesting of liver and pancreas tissues. The animal experiments were approved by the Institutional Animal Care and Use Committee of Southwest Hospital, Chongqing, China.

### Supplemental materials and methods

The Supplemental Materials are provided in **Table S1**. And the Supplemental Methods, such as the microarray analysis, RNA sequencing analysis, DNA methylation analysis, RNA ISH, fluorescence in situ hybridization (FISH), RNA immunoprecipitation (RIP), Chromatin immunoprecipitation (ChIP) and immunoprecipitation (IP), are provided in **Table S2**. The workflow of the clinical samples used in the present study is provided in **Figure S1**.

## Results

### LINC00261 is downregulated in PC tissue and is associated with survival

**Figure 1:**
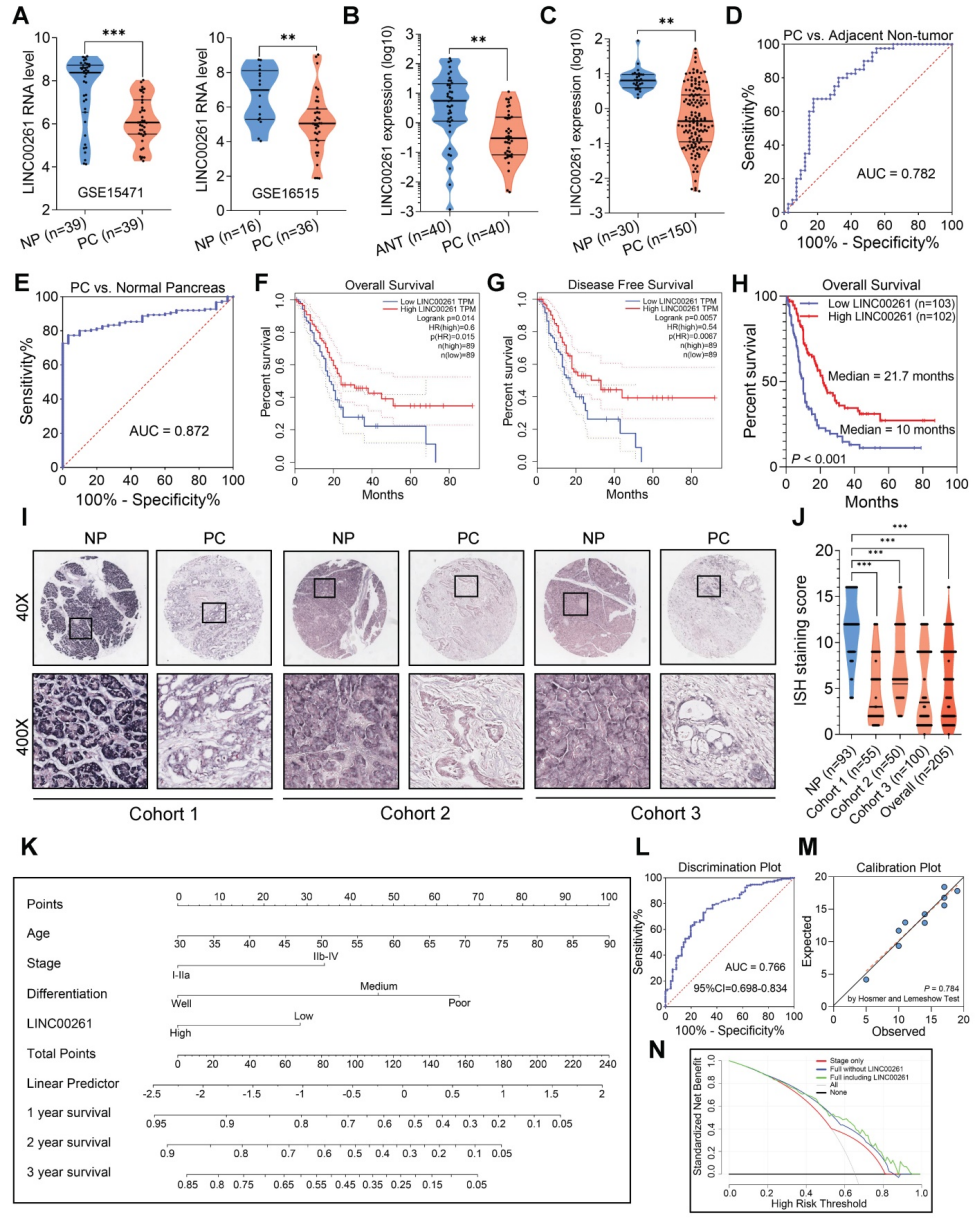
** LINC00261 is downregulated in PC tissue and is associated with survival. (A)** Expression profile of LINC00261 in the two GEO datasets (GSE15471 and GSE16515). **(B)** LINC00261 expression was assessed by qRT-PCR in paired pancreatic cancer (PC) and adjacent nontumor (ANT) tissues. **(C)** LINC00261 expression was assessed by qRT-PCR in PC and normal pancreas (NP) tissues. **(D)** Receiver operating characteristic (ROC) curve analysis was performed on paired PC patient tissues based on LINC00261 mRNA expression. **(E)** ROC curve analysis was performed on PC and NP tissues based on LINC00261 mRNA expression. **(F)** Kaplan-Meier analysis of overall survival of PC patients based on LINC00261 TCGA data (PC, N=178, the study group was dichotomized using the median expression value of LINC00261, dotted lines indicate the corresponding 95% confidence intervals for the two curves, analyzed in GEPIA). **(G)** Kaplan-Meier analysis of disease-free survival of PC patients based on LINC00261 TCGA data (PC, N=178, dotted lines indicate the corresponding 95% confidence intervals for the two curves, analyzed in GEPIA). **(H)** Kaplan-Meier analysis of overall survival of PC patients based on LINC00261 ISH score data (PC, N=205). **(I)** Representative images of LINC00261-positive staining are shown in the three independent PC and NP cohorts. **(J)** LINC00261 expression was assessed by ISH in PC tissue microarrays combining PC samples from three clinical centers and NP samples (Dots with the same ISH score may be overlapped in the figure). **(K)** Nomogram based on age, clinical stage, differentiation grade and LINC00261 expression level. **(L)** Validation of the discrimination ability of the nomogram using the bootstrap corrected area under curve (AUC) and 95% confidence interval (CI)**. (M)** Validation of the calibration of the nomogram using the Hosmer-Lemeshow test.** (N)** Decision curve analysis comparing the standardized net benefit of the following three models: model including tumor stage only; nomogram (as showed in Figure 1K) constructed in the present study but not including LINC00261; nomogram constructed in the present study. (*P < 0.05, **P < 0.01, ***P < 0.001).

To investigate the lncRNA expression profiles in PC, we applied data mining techniques to GEO datasets (GSE15471 and GSE16515) to identify the differentially expressed lncRNAs between PC and NP tissues. Through integrative gene microarray analysis, we screened 6 upregulated lncRNAs and 15 downregulated lncRNAs in PC tissues (**Figure S2A-B**). In our previous work, we studied the upregulated lncRNAs 21. Thus, we focused on LINC00261 among the 15 downregulated lncRNAs because it was the only annotated lncRNA associated with both the overall survival and disease-free survival of PC patients according to GEPIA database analysis 22 (**Figure 1A & Figure S2C-D**). We further validated LINC00261 expression by qRT-PCR in PC tissues, paired adjacent nontumor tissues, unpaired adjacent nontumor tissues and NP tissues. LINC00261 expression was markedly downregulated in PC tissues compared with nontumor and NP tissues (**Figure 1B**-**C**), consistent with the microarray analysis results. Receiver operating characteristic (ROC) curve analysis performed in parallel revealed the potential diagnostic value of this lncRNA in distinguishing the paired tumors from the adjacent nontumor tissues and distinguishing the tumors from NP tissues (**Figure 1D**-**E**). Overall survival and disease-free survival were analyzed in TCGA data 22 (**Figure 1F**-**G**). We further detected LINC00261 expression in tissue microarrays via RNA ISH. Samples were stratified into high and low expression groups according to the ISH staining score standard (**Table S2**). Survival analysis based on the ISH results revealed that LINC00261 was positively associated with overall survival. Patients in the high LINC00261 expression group had longer median survival times (21.7 months) than those in the low LINC00261 expression group (10 months, **Figure 1H**). LINC00261 expression was markedly downregulated in PC tissues, but was expressed at high levels in NP tissues (**Figure 1I**-**J**). These results demonstrate that LINC00261 is downregulated in PC tissue and is positively associated with survival.

### LINC00261 expression is correlated with progression and poor prognosis in PC

We then analyzed the association between LINC00261 expression and clinical features using the PC samples from the three cohorts underwent ISH. LINC00261 expression was negatively correlated with the pathological differentiation grade, clinical stage and lymph node metastasis in PC patients (all P < 0.05, **Table S3**). Univariate and subsequent multivariate Cox regression analysis revealed that LINC00261 was an independent protective factor for survival (**Table S4**). To expand the clinical significance of these results, we developed a nomogram based on the four independent covariates (age, clinical stage, differentiation grade and LINC00261 expression level) to predict the survival probability for PC patients (**Figure 1K**). The nomogram showed good performance in identifying outcome events (**Figure 1L**, bootstrap corrected AUC=0.766, 95%CI=0.698-0.834). In addition, the Hosmer-Lemeshow test indicated that the calibration of the nomogram was good (**Figure 1M,** P=0.784). The decision curve showed that if the threshold probability of survival is >20%, the nomogram added more net benefit than the treat-all-patients scheme or the treat-none scheme 23. In addition, the net benefit of the nomogram with LINC00261 included was higher than that of the nomogram without LINC00261 included at threshold probabilities ranging from 55% to 95% (**Figure 1N**). These data demonstrate that LINC00261 is significantly associated with favorable clinical characteristics and is an independent protective factor for PC survival.

### LINC00261 induces cell cycle arrest and inhibits tumor cell proliferation, migration and invasion in vitro

**Figure 2:**
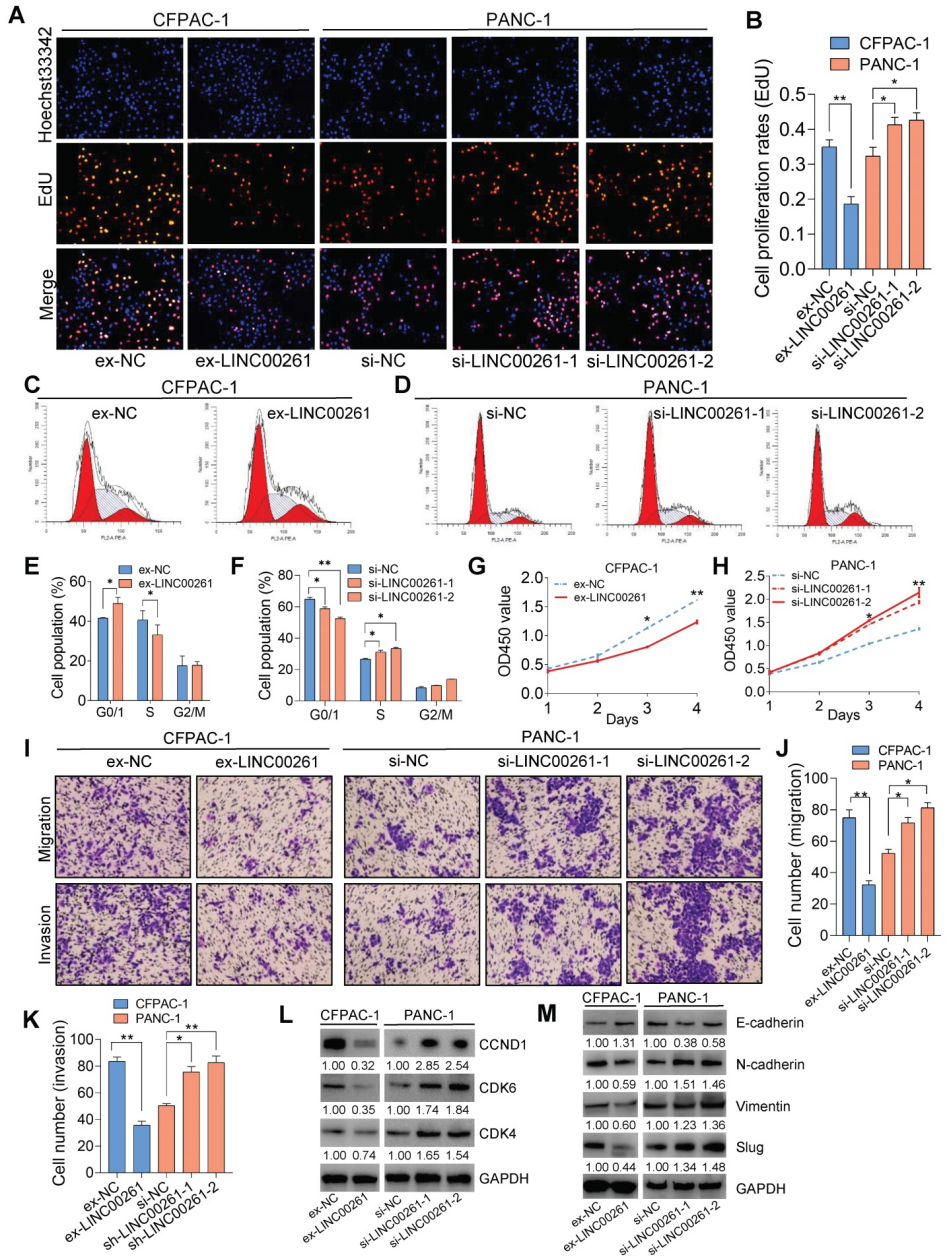
** LINC00261 significantly inhibits PC cell proliferation, migration and invasion *in vitro*. (A, B)** EdU assays were used to assess the cell proliferation ability. Histogram showing the proliferation rates of transfected cells in the corresponding groups. **(C, E)** Flow cytometric analysis showed that overexpression of LINC00261 significantly increased the proportion of CFPAC-1 cells in the G0/G1 phase and decreased that of cells in the S phase. **(D, F)** Flow cytometric analysis showed that knockdown of LINC00261 significantly decreased the proportion of PANC-1 cells in the G0/G1 phase and increased that of cells in the S phase.** (G, H)** CCK8 assays were used to assess the viability of transfected PC cells. **(I-K)** Transwell and Matrigel assays were used to assess PC cell migration and invasion abilities. Histogram showing the numbers of migrated and invaded PC cells. **(L)** WB analysis was performed to assess the expression of cell cycle-related markers. **(M)** WB analysis was performed to assess the expression of EMT-related markers (*P < 0.05, **P < 0.01, ***P < 0.001).

To investigate the effect of LINC00261 on the malignant phenotypes of PC cells, we first assessed LINC00261 expression in four PC cell lines and performed gain- and loss-of-function studies in PC cells by constructing a LINC00261 overexpression vector and LINC00261-siRNA (**Figure S3A-E**). LINC00261 overexpression induced cell cycle arrest and reduced cell proliferation, viability, migration and invasion relative to the corresponding properties in control cells, as shown by the results of the EdU assay, flow cytometric analysis, CCK8 assay, Transwell assay (migration) and Matrigel assay (invasion) (**Figure 2A-K & Figure S4A-F**). Furthermore, the Western blot (WB) results indicated that cell proliferation and the expression levels of the cell cycle-related markers CCND1, CDK4 and CDK6 were significantly decreased in LINC00261-overexpressing cells compared to control cells (**Figure 2L**). Simultaneously, the expression level of the epithelial-mesenchymal transition (EMT)-related epithelial marker E-cadherin was significantly increased, while those of N-cadherin, Vimentin and Slug were decreased (**Figure 2M**). In contrast, downregulation of LINC00261 enhanced cell cycle progression and increased cell proliferation, viability, migration and invasion relative to the corresponding properties in control cells (**Figure 2A-K**). WB results showed significantly increased expression levels of the cell cycle-related markers CDK4, CDK6 and CCND1 (**Figure 2L**) and a markedly decreased expression level of the EMT-related epithelial marker E-cadherin but increased expression levels of N-cadherin, Vimentin and Slug (**Figure 2M & Figure S4G**-**H**). These results demonstrate that LINC00261 plays an important role in inhibiting the proliferation and EMT-related invasion and migration of PC cells *in vitro*.

### LINC00261 suppresses tumor progression in vivo

**Figure 3:**
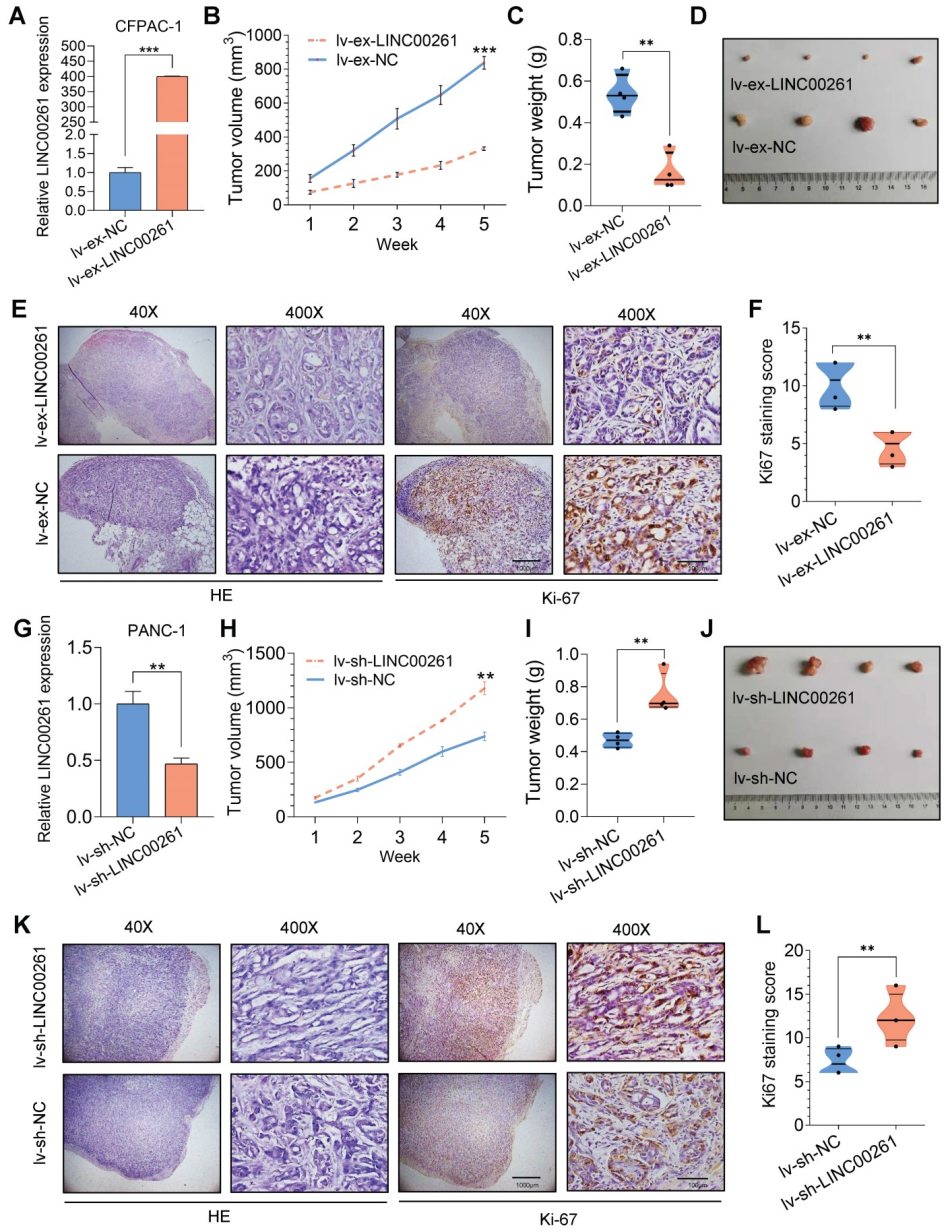
**LINC00261 influences tumor growth *in vivo*. (A)** qRT-PCR analysis was performed on LINC00261-overexpressing CFPAC-1 cells using a lentiviral system. **(B)** LINC00261 overexpression inhibited PC cell proliferation *in vivo*. CFPAC-1 cells with stable expression of LINC00261 and control cells were subcutaneously injected into 4- to 6-week-old female nude mice. The chart shows the tumor volume as measured each week in the control and LINC00261 overexpression groups. **(C)** Histogram indicating the mean tumor weights 5 weeks after inoculation in the control and LINC00261 overexpression groups. **(D)** Images of tumor lesions in the control and LINC00261 overexpression groups. **(E)** Representative images of hematoxylin and eosin (HE) staining and Ki67 immunostaining of tumor samples from mice in different groups. **(F)** The Ki67 staining score was assessed in the control and LINC00261 overexpression groups. **(G)** qRT-PCR analysis was performed on LINC00261 knockdown PANC-1 cells using a lentiviral system. **(H)** Knockdown of LINC00261 promoted PC cell proliferation *in vivo*. Chart showing the tumor volume as measured each week in the control and LINC00261 knockdown groups. **(I)** Histogram indicating the mean tumor weights 5 weeks after inoculation in the control and LINC00261 knockdown groups.** (J)** Images of tumor lesions in the control and LINC00261 knockdown groups. **(K)** Representative images of HE staining and Ki67 immunostaining of tumor samples from mice in different groups. **(L)** Ki67 staining score in the control and LINC00261 knockdown groups (scale bars, 1000 µm and 100 µm). (*P < 0.05, **P < 0.01, ***P < 0.001).

**Figure 4:**
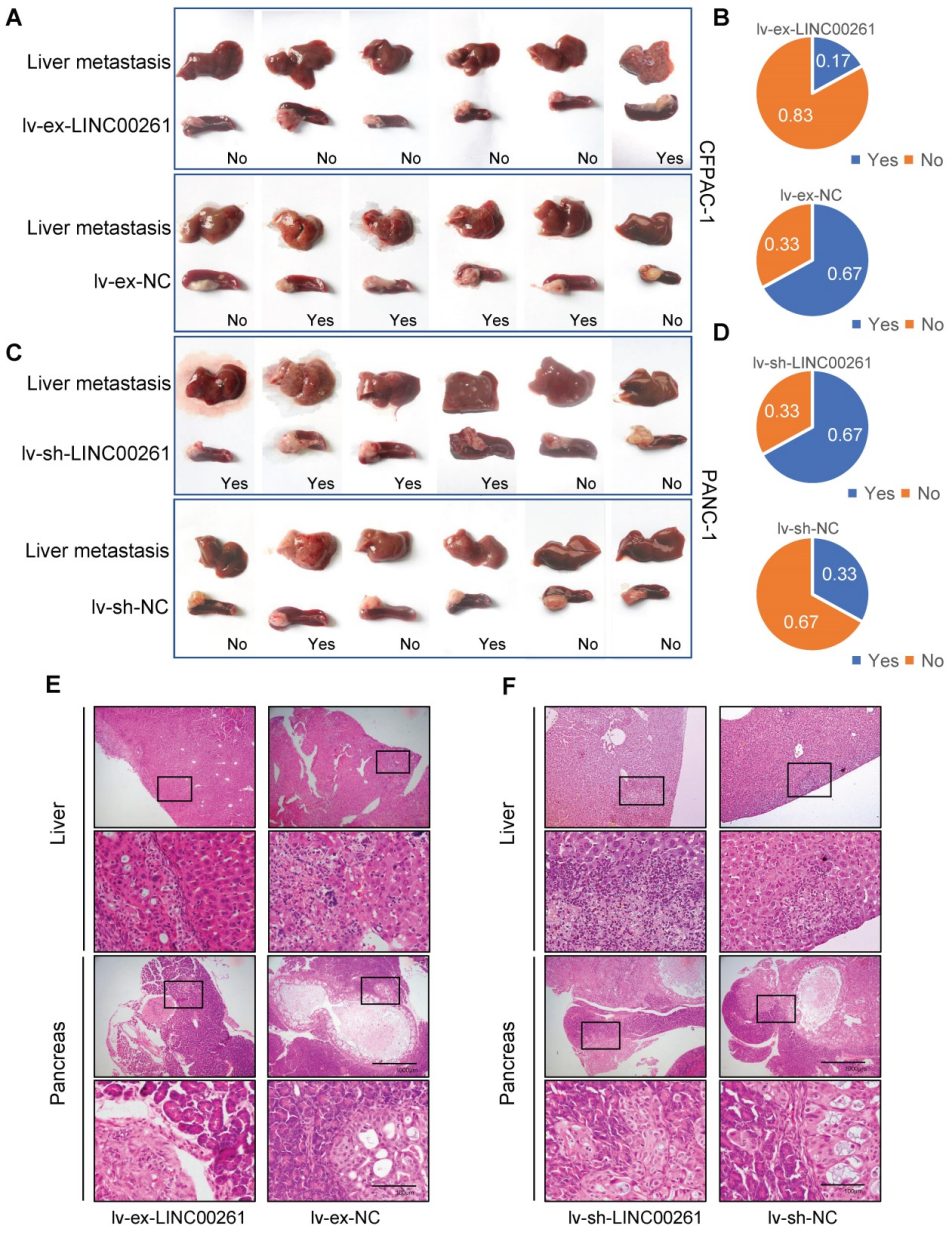
** LINC00261 influences tumor liver metastasis *in vivo*. (A)** LINC00261 overexpression inhibited liver metastasis of CFPAC-1 cells (derived from liver metastatic lesions of pancreatic ductal adenocarcinoma) *in vivo*. Images of micrometastases on the liver surface and pancreas xenograft in mice in the LINC00261 overexpression group and the control group. **(B)** Chart indicating the proportion of mice with liver metastases in the two groups. **(C)** Knockdown of LINC00261 promoted liver metastasis of PANC-1 cells (derived from in site lesions of pancreatic ductal adenocarcinoma) *in vivo*. Images of micrometastases on the liver surface in mice in the LINC00261 knockdown group and the control group. **(D)** Chart indicating the proportion of mice with liver metastasis in the two groups. **(E and F)** Representative images of in site pancreatic and liver metastatic lesions stained with HE in different groups (scale bars, 1000 µm and 100 µm). (*P < 0.05, **P < 0.01, ***P < 0.001).

We then evaluated the inhibitory effect of LINC00261 on PC progression *in vivo*. First, we established cell lines with stable LINC00261 upregulation and downregulation via lentiviral transfection. Successful overexpression and knockdown of LINC00261 in CFPAC-1 and PANC-1 cells were validated by qRT-PCR (**Figure 3A, G**). To ascertain the effect of LINC00261 on tumorigenicity *in vivo*, we subcutaneously transplanted the transfected cells into the dorsal region of nude mice. Overexpression and knockdown of LINC00261 affected tumor growth *in vivo* (**Figure 3B-F & H-L**). Then, stably transfected PC cells were injected into the distal pancreatic tissues of nude mice to assess whether LINC00261 influences liver metastasis of PC cells *in vivo*. Obviously, one mice (17%) in the LINC00261 overexpression group exhibited liver metastasis, while four mice (67%) exhibited intrahepatic metastasis in the LINC00261 knockdown group (**Figure 4A-F**). Taken together, our data demonstrate that LINC00261 acts as a suppressor of pancreatic tumor progression *in vivo.*

### c-Myc is a key downstream target of LINC00261

**Figure 5:**
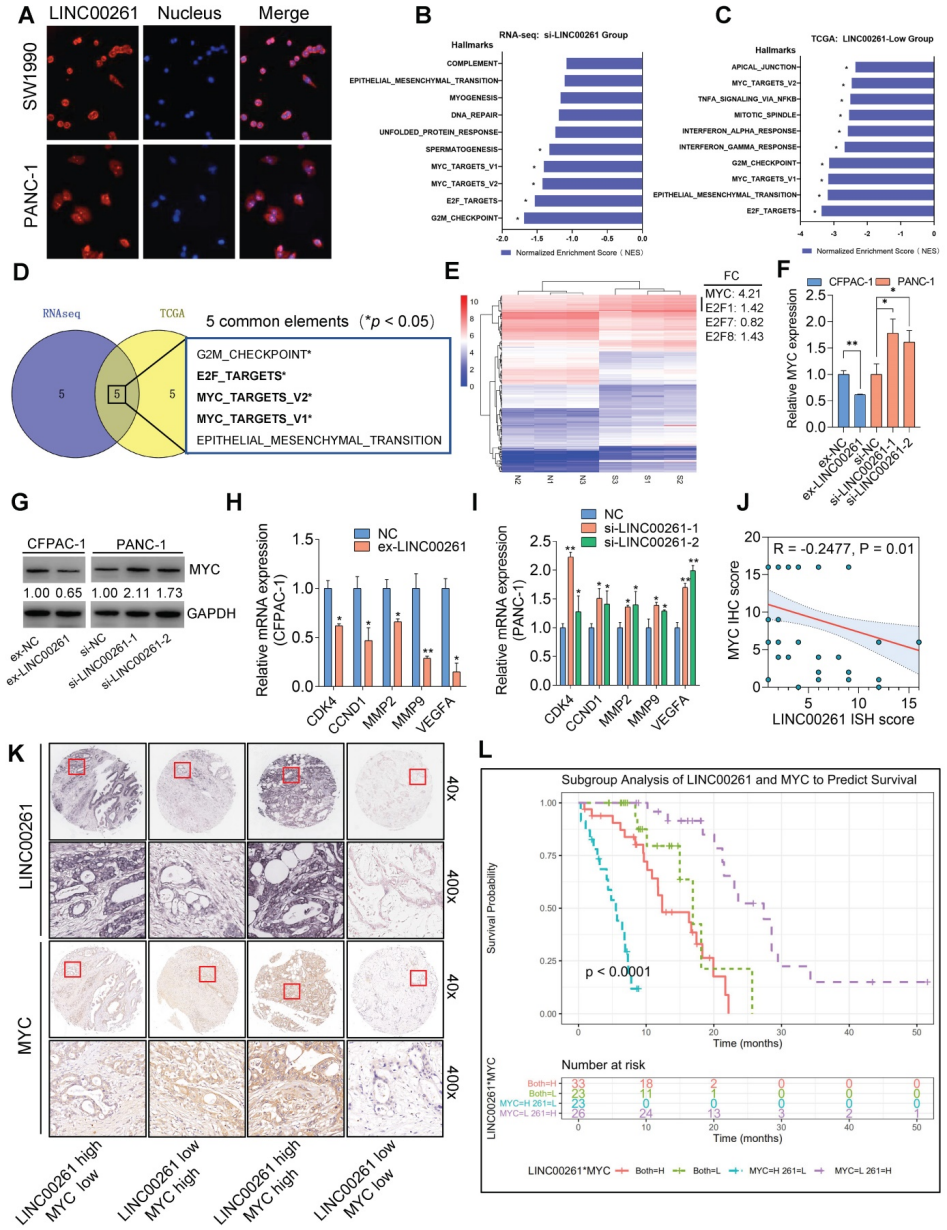
** c-Myc is a critical downstream target of LINC00261. (A)** FISH analysis showing the subcellular localization of LINC00261 in PANC-1 and SW1990 cells. **(B and C)** The top 10 enriched pathways in cells with LINC00261 silencing and patients with low LINC00261 expression identified by GSEA focused on a set of hallmark signaling pathways. Data are summarized based on the normalized enrichment score (NES). **(D)** Venn diagram of common enrichment pathways between the two groups (*NOM P-value <0.05**). (E)** Heatmap of differentially expressed downstream genes in LINC00261 knockdown PANC-1 cells (S1-3) and the corresponding control cells (N1-3) (P<0.05). **(F)** c-Myc gene expression levels, as analyzed by qPCR, in the LINC00261 knockdown group and the LINC00261 overexpression group. **(G)** c-Myc protein expression levels, as analyzed by WB, in the LINC00261 knockdown group and the LINC00261 overexpression group. **(H)** Expression levels of c-Myc-related downstream genes, as analyzed by qPCR, in LINC00261-overexpressing CFPAC-1 cells and control cells. **(I)** Expression levels of c-Myc-related downstream genes, as analyzed by qPCR, in LINC00261-knockdown PANC-1 cells and control cells. **(J)** Spearman correlation analysis of the association between LINC00261 and c-Myc. Fisher's test was performed in parallel (P=0.002, MYC and LINC00261 expressions were dichotomized using the median value)**. (K)** Representative images of different staining intensities of ISH or IHC staining for LINC00261 and c-Myc in tissues (high: high expression; low: low expression). **(L)** Kaplan-Meier survival analysis of the four groups of PC patients stratified based on LINC00261 ISH data and c-Myc IHC data (*P < 0.05, **P < 0.01, ***P < 0.001).

Since the molecular function of lncRNA largely depends on its subcellular location 24, we performed FISH and found that LINC00261 was expressed in both the nucleus and cytoplasm, indicating that it may regulate a variety of biological processes (**Figure 5A**). To investigate the downstream pathway of LINC00261 in PC, we performed RNA-seq on two lines of PANC-1 cells: cells with LINC00261 downregulation and control cells (each cell line was biologically repeated in triplicate). Then, we performed Gene Set Enrichment Analysis (GSEA, Broad Institute) on our RNA-seq dataset (by dividing the transfected PC cells into the si-LINC00261 group and the si-NC group) and analyzed the top 10 enriched pathways (**Figure 5B**). We next performed the same analysis on the TCGA dataset (by dividing the patients into the LINC00261-high group and the LINC00261-low group) for independent validation (**Figure 5C**). The combined results of both analyses revealed that the differential expression of LINC00261 was statistically significantly related to Myc, E2F and G2M hallmarks (**Figure 5D**). We selected the Myc and E2Fs because they are important transcription factors in PC progression 25 and they both could regulate the cell cycle, migration and invasion from KEGG database (Pathways in cancer). The G2M was excluded as the change in cell cycle is not obvious in **Figure 2C-D**. Subsequent cluster analysis showed that LINC00261 knockdown led to alterations in gene expression, including in the Myc and E2F families. Importantly, the gene expression analysis indicated that Myc was differentially expressed with a higher fold change than E2F related genes; thus, we focus on the gene Myc (**Figure 5E & Figure S5A**). The correlation analyses of TCGA data revealed that LINC00261 was negatively correlated with Myc. Combined with KEGG analysis, other known downstream targets of Myc, such as CDK4, CCND1, MMPs and VEGFA 26-30, were also negatively correlated with LINC00261 (**Figure S5B**).

Importantly, Kaplan-Meier survival analysis of patient subgroups showed that the group with higher LINC00261 expression and lower Myc expression had the best prognosis (**Figure S5C**). These results suggest that Myc is a target of LINC00261. To further confirm this finding, we performed qPCR and WB analyses on PC cells with overexpression and downregulation of LINC00261. c-Myc expression was negatively regulated by LINC00261 (**Figure 5F**-**G & Figure S5D-E**). Similarly, the expression levels of CDK4, CCND1, MMP2, MMP9 and VEGFA were decreased when LINC00261 was overexpressed (**Figure 5H**). In contrast, the expression levels of these genes were increased when LINC00261 was downregulated (**Figure 5I**). Then, the expression levels of LINC00261 and c-Myc were evaluated in microarrays containing tissues from the same group of patients. LINC00261 was also negatively associated with c-Myc in these PC tissues by the Spearman's test and the Fisher's test (**Figure 5J**-**K**). Furthermore, subgroup Kaplan-Meier analysis showed that patients with high LINC00261 expression and low c-Myc expression had the best prognosis (**Figure 5L**), consistent with our prior observations in the TCGA dataset. Taken together, these results demonstrate that c-Myc is negatively regulated by LINC00261 and is likely associated with the clinical prognosis of PC patients via interaction with LINC00261.

### Regulation of cell proliferation, migration and invasion by LINC00261 is dependent on c-Myc

**Figure 6:**
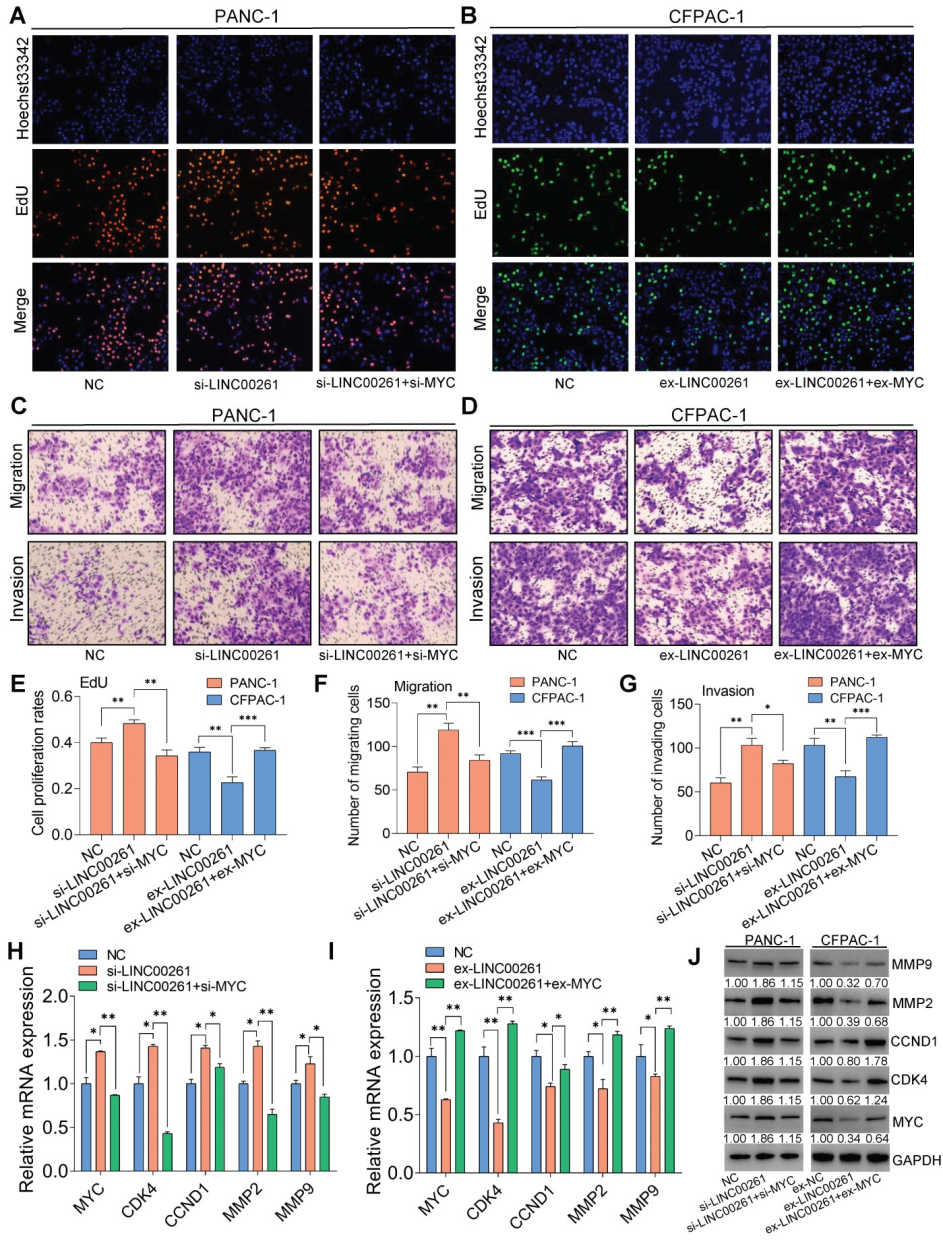
** LINC00261 functions via the transcription factor c-Myc in PC. (A, C)** Knockdown of c-Myc in cells with low LINC00261 expression. EdU, Transwell assays and Matrigel assays were used to assess proliferation, migration and invasion in NC, si-LINC00261 and si-LINC00261+si-c-Myc PANC-1 cells. **(B, D)** Overexpression of c-Myc in LINC00261-overexpressing cells. EdU, Transwell and Matrigel assays were used to assess proliferation, migration and invasion in three groups of CFPAC-1 cells: NC, ex-LINC00261 and ex-LINC00261+ex-c-Myc. **(E)** Histogram showing the proliferation rates of cotransfected PANC-1 and CFPAC-1 cells in the three groups. **(F, G)** Histogram showing the number of migrated and invaded cotransfected PANC-1 and CFPAC-1 cells in the three groups. **(H)** The mRNA levels of c-Myc and its related downstream molecules, as assessed by qRT-PCR, in the NC, si-LINC00261 and si-LINC00261+si-c-Myc groups. **(I)** The mRNA levels of c-Myc and its related downstream molecules, as assessed by qRT-PCR, in the NC, ex-LINC00261 and ex-LINC00261+ex-c-Myc groups. **(J)** The protein levels of c-Myc and its related downstream molecules, as assessed by WB analysis, in the NC, si-LINC00261 and si-LINC00261+si-c-Myc groups. The protein levels of c-Myc and its related downstream molecules, as assessed by WB analysis, in the NC, ex-LINC00261 and ex-LINC00261+ex-c-Myc groups. (*P < 0.05, **P < 0.01, ***P < 0.001).

Next, we used EdU, Transwell and Matrigel assays to investigate whether the LINC00261-induced inhibition of cell proliferation, migration and invasion is dependent on c-Myc. Compared with control cells, PC cells transfected with LINC00261-specific siRNA exhibited enhanced malignant behaviors, namely, proliferation, migration and invasion. However, these increases were significantly reversed in cells cotransfected with LINC00261-specific siRNA, c-Myc-specific siRNA or a c-Myc inhibitor (**Figure 6A**, **C & Figure S6**). We inversely validated these results by overexpressing LINC00261 alone or with c-Myc. As expected, overexpression of LINC00261 inhibited the proliferation, migration and invasion of PC cells, while these inhibitory effects were reversed when c-Myc was overexpressed (**Figure 6B, D**). These functional assay results are summarized in the histograms (**Figure 6E-G**). The mRNA and protein expression levels of c-Myc downstream targets, including MMP9, MMP2, CCND1, and CDK4, were increased when LINC00261 was downregulated, and this effect was reversed when LINC00261 and c-Myc were simultaneously downregulated (**Figure 6H, J & Figure S7A-C**). In addition, the expression levels of these targets were decreased when LINC00261 was overexpressed, and this effect was reversed when LINC00261 and c-Myc were simultaneously overexpressed (**Figure 6I, J & Figure S7A-C**). Taken together, these results indicate that the inhibitory effects of LINC00261 on the proliferation, migration and invasion of PC cells are dependent on c-Myc.

### LINC00261 physically interacts with the p300/CBP complex to epigenetically repress c-Myc transcription

**Figure 7:**
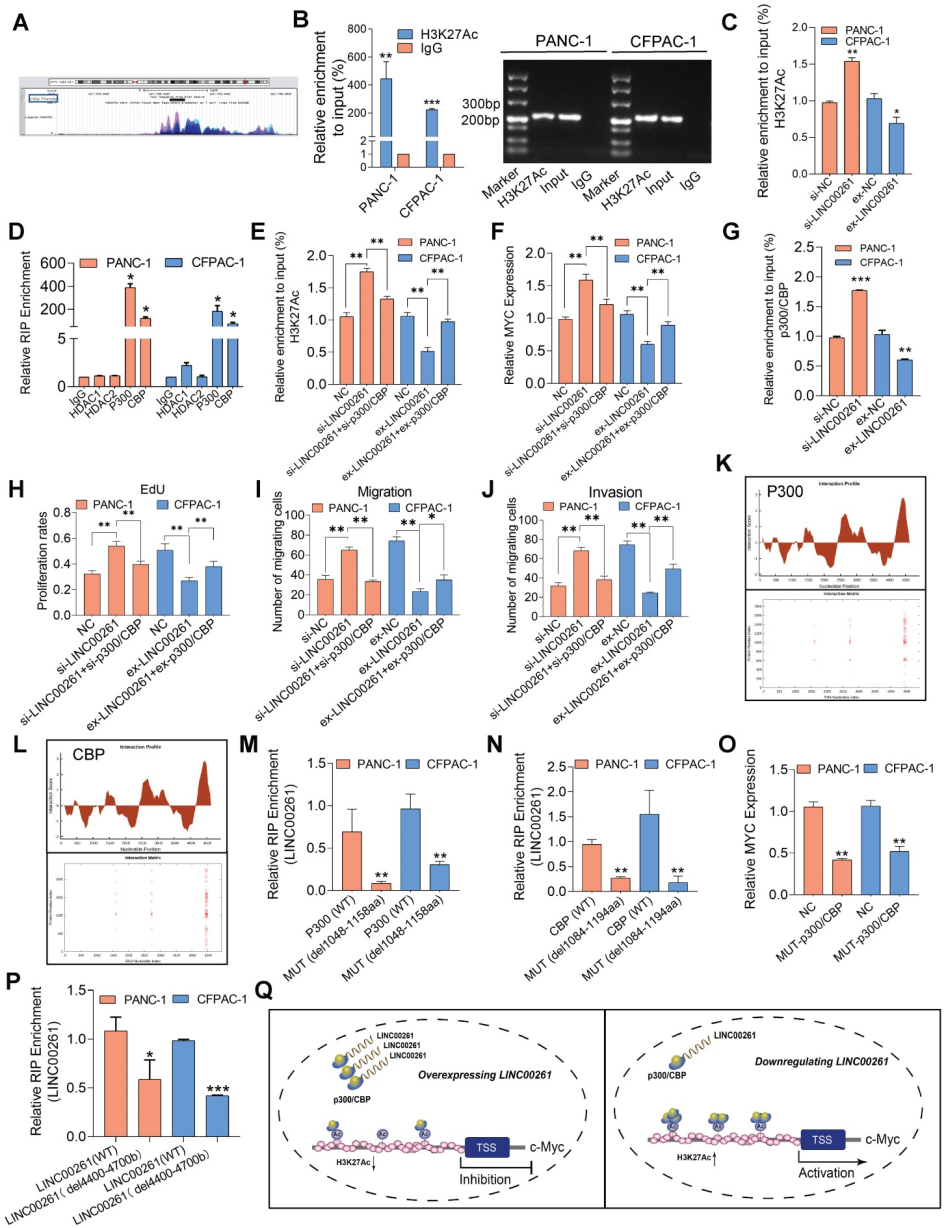
** LINC00261 inhibits c-Myc-driven malignant phenotypes via p300/CBP. (A)** H3K27 acetylation of the c-Myc promoter region in the UCSC genome database. **(B)** ChIP assays with an anti-H3K27Ac antibody or IgG were performed to verify the enrichment of H3K27Ac at binding sites in the c-Myc promoter in PC cells. The ChIP-PCR products in the c-Myc, input and IgG groups were evaluated by agarose gel electrophoresis. **(C)** ChIP assays with an anti-H3K27Ac antibody or IgG were performed in PC cells with LINC00261 knockdown, PC cells with LINC00261 overexpression and control cells. **(D)** RIP assays were performed to validate LINC00261 binding to related proteins in PANC-1 and CFPAC-1 cells. **(E)** ChIP assays with an anti-H3K27Ac antibody or IgG were performed in the three groups of PANC-1 cells (si-NC, si-LINC00261, si-LINC00261+si-p300/CBP) and in the three groups of CFPAC-1 cells (ex-NC, ex-LINC00261 and ex-LINC00261+ex-p300/CBP). **(F)** c-Myc gene expression levels were analyzed by qRT-PCR in the three groups of PANC-1 cells (si-NC, si-LINC00261, si-LINC00261+si-p300/CBP) and in the three groups of CFPAC-1 cells (ex-NC, ex-LINC00261 and ex-LINC00261+ex-p300/CBP). **(G)** ChIP assays with an anti-p300/CBP antibody or IgG were performed to verify the enrichment of p300/CBP at binding sites in the c-Myc promoter in PC cells with LINC00261 downregulation and overexpression. **(H)** Histogram showing the proliferation rates of cotransfected PC cells.** (I** and** J)** Histograms showing the number of migrated and invaded cotransfected PC cells. **(K and L)** The interaction profile, which represents the P300/CBP interaction score (Y-axis) relative to the LINC00261 RNA sequence (X-axis), provides information about the region most likely to be bound by the protein (up); The interaction matrix, which shows a heatmap of the P300/CBP(Y-axis) and LINC00261 RNA (X-axis) regions. The red shading in the heatmap indicates the interaction score of a single amino acid and nucleotide pair (down). **(M and N)** RIP assays with an anti-Flag antibody or IgG were performed to verify the LINC00261 enrichment in PANC-1 and CFPAC-1 cells transfected with the p300 vector (WT) or mutant p300 vector (del-1048-1158aa) and the CBP vector (WT) or the mutant CBP vector (del-1084-1194aa). All plasmids were labeled with 3-flag.** (O)** The mRNA levels of c-Myc was assessed by qRT-PCR, in the NC and mut-p300/CBP groups. **(P)** RIP assays were performed to validate p300/CBP binding sites of LINC00261 region in PANC-1 and CFPAC-1 cells transfected with LINC00261 (WT) and mutant LINC00261 vector (del-4400bp-4700bp). **(Q)** A schematic model of the mechanism underlying the role of LINC00261 in PC progression (*P < 0.05, **P < 0.01, ***P < 0.001).

A previous study suggested that lncRNAs epigenetically mediate gene transcription by modifying histone acetylation or methylation levels via interaction with acetylases 31 or methylases 32. Considering that c-Myc transcription is regulated by histone acetylation as a classical mechanism 33, we sought to determine whether this mechanism was involved in LINC00261-mediated regulation. First, we focused on H3K27 acetylation (H3K27Ac) in the c-Myc gene promoter and found that the c-Myc promoter region can undergo H3K27Ac modification, according to analysis of the UCSC genome database (http://genome.ucsc.edu/, **Figure 7A**). We constructed the WT c-Myc promoter vector and performed reporter gene assays. LINC00261 overexpression significantly decreased the luciferase activity, and the opposite pattern was observed in the LINC00261 knockdown group (**Figure S7D**). We performed ChIP-PCR on PC cells, and the results validated high enrichment of H3K27Ac at the c-Myc promoter region (**Figure 7B**). We further investigated whether LINC00261 modifies the H3K27Ac level at the c-Myc gene promoter. Consistent with our prediction, the H3K27Ac level at the c-Myc promoter was decreased after LINC00261 overexpression. In contrast, the H3K27Ac level was increased after LINC00261 downregulation (**Figure 7C**).

To further investigate whether LINC00261 modifies the H3K27Ac level in the c-Myc promoter by interacting with acetylase or deacetylases, we first used an online tool to predict possible LINC00261-protein interactions (http://pridb.gdcb.iastate.edu/RPISeq/index.html, **Figure S7E**). The results of a subsequent RNA immunoprecipitation (RIP) assay verified that LINC00261 could bind to the acetylase p300/CBP but not to HDAC1 or HDAC2 (**Figure 7D**). p300/CBP is an important acetylase complex that can regulate the H3K27Ac level 34. We speculated that LINC00261 modifies H3K27Ac through the p300/CBP complex. The ChIP-PCR results showed that overexpression of LINC00261 decreased the H3K27Ac level at the c-Myc promoter, while this decrease was significantly reversed in cells cotransfected with the LINC00261 overexpression vector and the p300/CBP overexpression vector. In contrast, downregulation of LINC00261 in PANC-1 cells increased the H3K27Ac enrichment at the c-Myc promoter, and this increase was significantly reversed in cells cotransfected with LINC00261-specific siRNA and p300/CBP-specific siRNA (**Figure 7E**). c-Myc mRNA expression was validated by qPCR, and similar results were observed (**Figure 7F**). Furthermore, LINC00261 overexpression decreased but LINC00261 downregulation increased the binding of p300/CBP to the c-Myc promoter (**Figure 7G**).

These results indicated that LINC00261 regulates H3K27Ac by interacting with p300/CBP and obstructing its binding to the acetylation site in the c-Myc promoter. Besides, we used EdU, Transwell and Matrigel assays to investigate whether the inhibitory effects of LINC00261 on cell proliferation, migration and invasion are dependent on p300/CBP. The inhibition of these malignant behaviors in CFPAC-1 cells induced by LINC00261 overexpression was reversed by cotransfection of the p300/CBP-specific overexpression vector. In contrast, the enhancement of these malignant behaviors in PANC-1 cells induced by downregulation of LINC00261 was reversed after cotransfection with p300/CBP-specific siRNA (**Figure 7H-J & Figure S7F-I**).

### LINC00261 physically binds to the bromo domain of p300/CBP

To further clarify the interacting mechanism of LINC00261 and p300/CBP, we first investigated the potential binding region using the online algorithm CatRAPID (http://service.tartaglialab.com/page/catrapid_group), which rapidly predicts RNA-protein interactions and domains to evaluate the interaction tendency based on secondary structures, hydrogen bonding, and molecular interatomic forces. CatRAPID analysis showed that the 1048 aa-1158 aa region of p300 and the 1084 aa-1194 aa region of CBP are the most likely regions bound by LINC00261 (**Figure 7K**-**L**). Importantly, these regions contain critical protein domains, i.e., bromo domains (Human Protein Reference Database). The bromo domain has been reported to be an important functional structure for p300/CBP acetylation 35-37; thus, we speculated that LINC00261 inhibits the effect of p300/CBP by interacting with this domain. We constructed mutant p300/CBP expression plasmids (**Figure S8**) and evaluated the interaction of mutant p300/CBP with LINC00261 in PC cells and observed lower enrichment in cells transfected with either the mutant p300 vector (del-1048-1158aa) or the mutant CBP vector (del-1084-1194aa) group compared with cells transfected with the WT constructs (**Figure 7M-N**). Furthermore, we found that c-Myc expression was weakened in PC cells transfected with the mutant p300/CBP vector group compared with control (**Figure 7O**). In addition, we constructed a mutant LINC00261 vector according to the predicted region to identify the p300/CBP binding sites of LINC00261 region (del-4400bp-4700bp). The RIP assays showed minimal enrichment in the mutant LINC00261 group compared with the WT group (**Figure 7P**), consistent with our prior CatRAPID prediction. These findings demonstrate that LINC00261 directly interacts with the bromo domain of p300/CBP and participated in the regulation of c-Myc.

Taken together, these results demonstrate that LINC00261 physically interacts with the bromo domain of p300/CBP and prevents p300/CBP from binding to the acetylation site, which in turn decreases the H3K27Ac level and influenced the c-Myc expression (**Figure 7Q**).

### High methylation levels of the LINC00261 promoter were observed in PC patients and correlated with poor prognosis

**Figure 8:**
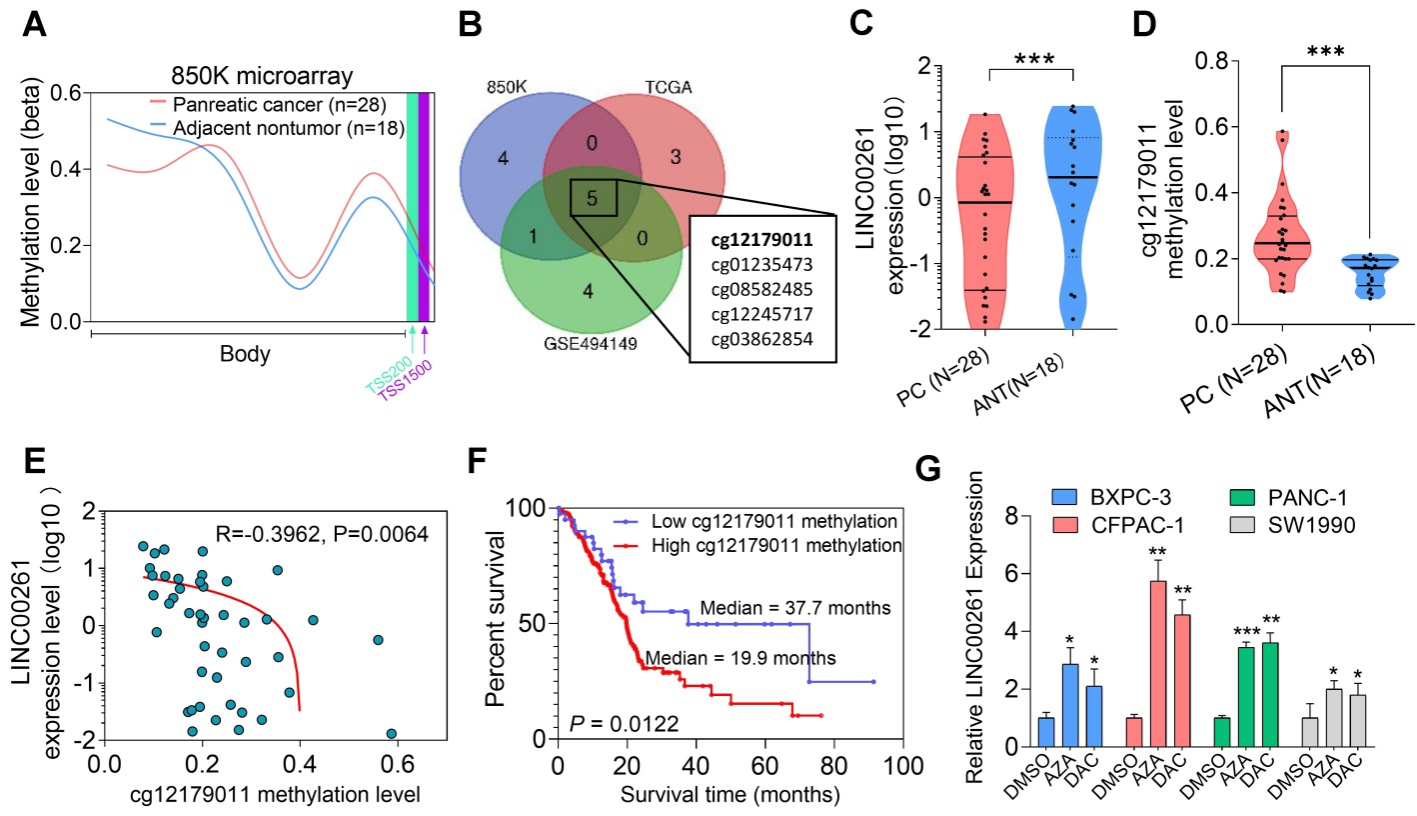
** High methylation levels of the LINC00261 promoter are observed in PC patients and are correlated with poor prognosis. (A)** Methylation levels of LINC00261 in the gene body and the promoter region in PC (N=28) and ANT tissues (N=18). **(B)** Venn diagram of significant differential methylation sites (CG) in LINC00261 in the three GEO datasets. The cg12179011 was in the promoter region, others were in the gene body. **(C)** LINC00261 expression as assessed by qRT-PCR in PC and ANT tissues. **(D)** Validation of the methylation level (cg12179011) in PC and ANT tissues by pyrosequencing. **(E)** Correlation analysis of LINC00261 expression and the cg12179011 methylation level. **(F)** Kaplan-Meier analysis of overall survival of PC patients stratified by the methylation level (cg12179011, the study group was dichotomized using the cg-value:1.5) using TCGA data. **(G)** LINC00261 expression in PC cells after treatment with DAC and AZA (*P < 0.05, **P < 0.01, ***P < 0.001).

High levels of promoter methylation might cause downregulation of lncRNAs in cancer, as previously described 38. To explore whether similar upstream mechanisms are involved in the regulation of LINC00261, we used Methylation EPIC BeadChip data (850K microarray) to evaluate the methylation level of the LINC00261 promoter. The methylation levels of LINC00261 in the gene body and promoter region were evaluated in PC and NP tissues, and we found that the methylation level of LINC00261 in PC tissues was higher than that in NP tissues (**Figure 8A**). Furthermore, the only methylation locus cg1279011 in the LINC00261 promoter was highly methylated in the three cohorts (**Figure 8B**). Next, we evaluated the expression level of LINC00261 and the methylation level of cg1279011 and observed that LINC00261 was downregulated in PC tissues compared to ANT tissues. Pyrosequencing analysis revealed that the methylation level of cg1279011 was higher in PC tissues than in ANT tissues (**Figure 8C-D**). Moreover, the LINC00261 expression and cg1279011 methylation levels were negatively correlated (**Figure 8E**). Patients were stratified into the high- and low-methylation-level groups, and patients in the high-methylation-level group had significantly shorter overall survival times (19.9 months) than those in the low-methylation-level group (37.7 months, **Figure 8F**). Next, we treated PC cells with azacitidine (AZA) and decitabine (DAC) and found that the expression level of LINC00261 in the BXPC-3, CFPAC-1, PANC-1 and SW1990 cell lines increased significantly (**Figure 8G**). Taken together, these results demonstrate the methylation level of LINC00261 is high in PC cells and that this high methylation level is associated with the prognosis of PC patients. In addition, this high methylation level might cause LINC00261 downregulation in PC.

### Targeted demethylation of the LINC00261 promoter inhibits the progression of PC

**Figure 9:**
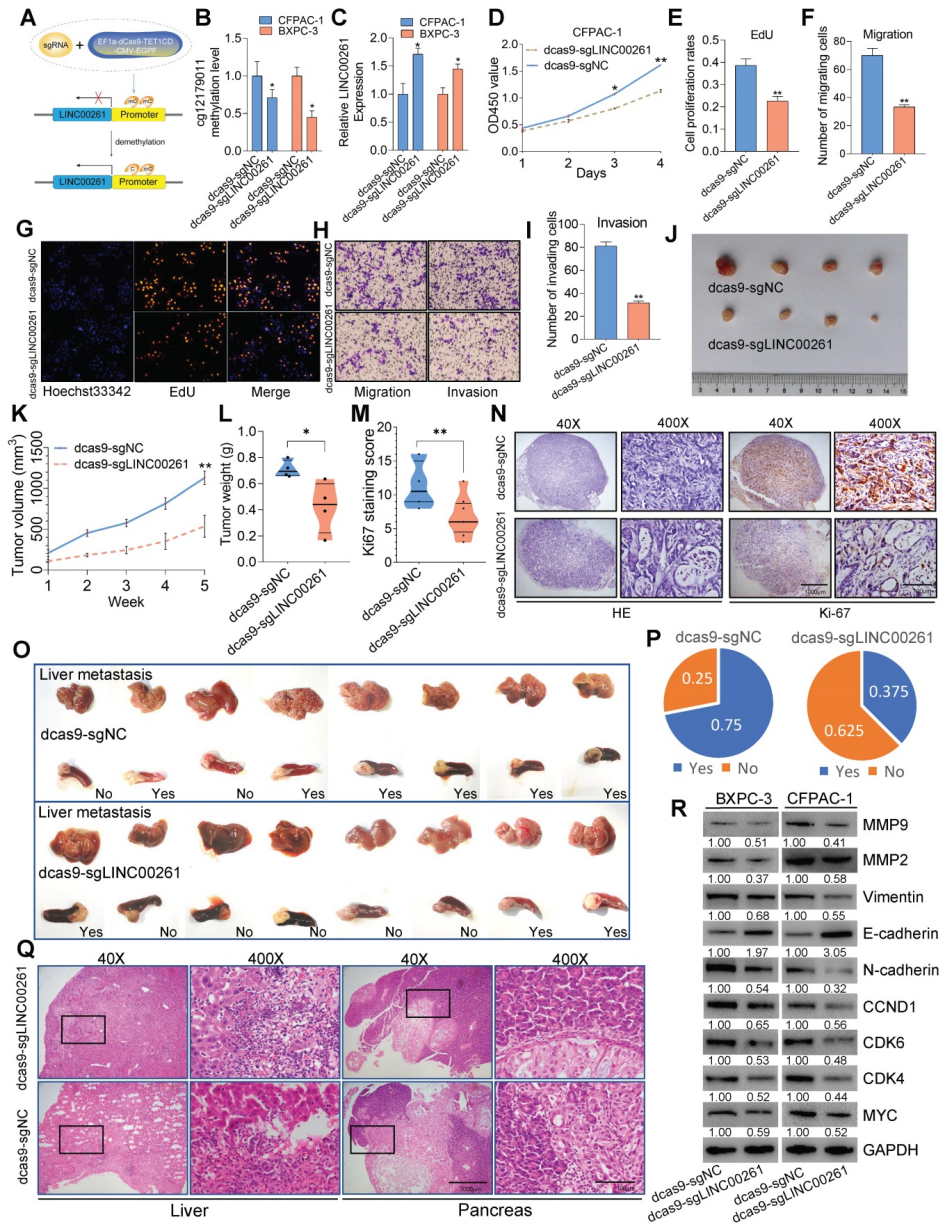
** Targeted demethylation of the LINC00261 promoter inhibits the progression of PC. (A)** Schematic of the targeted demethylation strategy via EF1a-Dcas9-Tet1CD-CMV-EGFP and sgRNA-LINC00261. **(B)** PC cells with stable expression of EF1a-Dcas9-Tet1CD-CMV-EGFP were generated (dCas9-PC cells). Then, genomic DNA extracted from PC cells was subjected to pyrosequencing after transfection. The methylation level of cg12179011 is shown (sgNC: no sgRNAs targeting LINC00261 sites). **(C)** LINC00261 expression was determined by qPCR after sgLINC00261 and sgNC transfection.** (D, E and G)** EdU and CCK8 assays were performed to determine the growth of dCas-CFPAC-1 cells transfected with sgLINC00261 and sgRNA-NC. The cell proliferation rate was calculated and analyzed. **(F, H and I)** Transwell and Matrigel assays were used to assess tumor cell migration and invasion after transfection. The migrated and invaded cells were counted and analyzed. **(J)** Antitumor effect of targeted demethylation of LINC00261 *in vivo*. Images of tumor lesions in the sgRNA-LINC00261 and sgRNA-NC groups. **(K)** Chart showing the tumor volume as measured each week after the injection of dCas-CFPAC-1 cells stably transfected with sgRNA-LINC00261 and sgRNA-NC vectors. **(L)** Histogram indicating the mean tumor weights 5 weeks after inoculation. **(M** and** N)** Representative images of HE staining and Ki67 immunostaining of tumor samples from mice in different groups. The Ki67 staining score was assessed. **(O)** Targeted demethylation inhibited liver metastasis of PC cells *in vivo*. Images of micrometastases on the liver surface in the sgRNA-LINC00261 and sgRNA-NC groups after the injection of dCas-CFPAC-1 cells stably transfected with sgRNA-LINC00261 and sgRNA-NC vectors**. (P)** Chart indicating the ratio of mice with liver metastasis in the two groups. **(Q)** Representative HE staining of pancreas and liver metastatic lesions (scale bars, 1000 µm and 100 µm). **(R)** Expression levels of EMT- and c-Myc-related downstream genes, as analyzed by WB, in the sgRNA-LINC00261 and sgRNA-NC groups (*P < 0.05, **P < 0.01, and ***P < 0.001).

To validate the relationship between LINC00261 promoter methylation and PC phenotypes, a CRISPR/dCas9 system 17 was used to enhance LINC00261 expression by demethylating its promoter (**Figure 9A**). We designed three sgRNAs targeting the cg1279011 site (**Figure S9A**). After transfection of PC cells with EF1a-Dcas9-Tet1CD-CMV-EGFP/sgLINC00261, we used pyrosequencing analysis to validate the methylation level of cg1279011 and found that it was decreased in CFPAC-1 and BXPC-3 cells (**Figure 9B**). In addition, the expression levels of LINC00261 in BXPC-3 and CFPAC-1 cells were increased (**Figure 9C**). Off-target effects are a major concern for the practical application of any Cas9-based technique; thus, evaluation of the off-target effects of the dCas9-based demethylation system was necessary. To this end, the top 15 potential off-target loci identified according to the CRISPOR web tool (http://crispor.tefor.net/) were selected for examination of whether their mRNA transcription levels were altered as a result of interference or random demethylation by CRISPR/dCas9. No significant differences in the mRNA expression levels of these potential off-target genes were found between cells transfected with EF1a-Dcas9-Tet1CD-CMV-EGFP/sgLINC00261 and control cells. Collectively, these results confirmed the specific effect of CRISPR/dCas9 editing on LINC00261 promoter demethylation (**Figure S9B**-**C**). We then performed EdU, Transwell and Matrigel assays and found that targeted demethylation of the LINC00261 promoter inhibited PC cell proliferation, viability, invasion and migration *in vitro* (**Figure 9D-I**). In addition, this targeted demethylation suppressed tumor growth (**Figure 9J-N**) and liver metastasis *in vivo* (**Figure 9O-Q**). WB analysis was further performed to evaluate the expression levels of protein markers of EMT and the cell cycle in PC cells. The expression levels of c-Myc, MMP9, MMP2, N-cadherin, vimentin, CCND1, CDK6 and CKD4 were decreased, while that of E-cadherin was increased (**Figure 9R**). Taken together, these data reveal that targeted demethylation of the LINC00261 promoter upregulates LINC00261 expression and inhibits PC progression.

## Discussion

In the present study, we demonstrated that LINC00261 is downregulated and has potential diagnostic value in PC. Furthermore, we found that LINC00261 represses c-Myc expression by interacting with and blocking the histone acetylase complex p300/CBP to decrease H3K27Ac enrichment at the c-Myc promoter. Downregulation of c-Myc had an important impact on its downstream target genes, eventually leading to the suppression of PC progression. In addition, the high methylation level of LINC00261 contributes to its low expression in PC, and targeted demethylation of the LINC00261 promoter restored the expression and function of LINC00261. These results might provide evidence helping us to further comprehend the molecular function of LINC00261 and inspiring novel diagnostic and therapeutic strategies for PC.

LINC00261 is an intergenic non-protein-coding RNA located on chromosome 20p11.21. In PC, LINC00261 has been suggested to be a prognostic marker 18, but the potential molecular mechanism remains largely unknown 19, 20. We validated the expression of LINC00261 from lncRNA microarray data by comparing data from the public cancer databases GEO and TCGA and showed that LINC00261 is downregulated in PC, consistent with the results of previous studies 18-20. More importantly, we found by analysis of tissue microarrays established from multicenter cohorts that LINC00261 is an independent protective factor for the survival of PC patients. To our knowledge, this study is the first to validate the prognostic value of LINC00261 in PC patients using multivariate analysis in multicenter cohorts with this sample size. Furthermore, the nomogram we developed using the cohort data has potential to guide clinical decisions. Although independent validation in external cohorts is needed to further validate its efficacy.

Competing endogenous RNA activity, such as miRNA sponging, is a well-known mechanism by which LINC00261 affects other cancers, as previously indicated 39. However, LINC00261 is 4924nt long, and we identified multiple protein-binding sites in its sequence, suggesting that LINC00261 might also act as a protein sponge by binding key regulatory molecules and further influencing the functions of their target genes such as CDK4 (**Figure S10**). The results suggest a possible mechanism that LINC00261 competitively binds to the c-Myc protein to block its transcriptional regulation on downstream targets. Although this mechanism requires further research, it suggests that the mechanism by which LINC00261 regulates c-Myc function might be complicated.

Considering the extreme importance of c-Myc in PC, many novel therapeutic approaches have been developed or are currently being developed to target c-Myc in order to suppress its function 40. However, studies reporting that c-Myc expression is epigenetically repressed by lncRNAs are scarce. A recent study has shown that the lncRNA PVT1 promotes liver cancer progression by disturbing EZH2-regulated histone methylation of the c-Myc promoter 32. Interestingly, our study revealed that LINC00261 physically interacts with the acetylase p300/CBP and blocks the binding of p300/CBP to the promoter region of c-Myc, leading to downregulation of c-Myc transcription. Importantly, through previous bioinformatic predictions and RIP experiments, we found that LINC00261 mainly bind to the bromo domain of p300/CBP, which mainly affects the biological function of acetylation. And the other transcription factors recruitment and binding domain is not in this region. Thus, we speculated that LINC00261 will not affect the binding of transcription factors to p300/CBP, but more experiments are needed to further verify this problem in the future (**Figure S11A**). These results indicate the complexity of lncRNA functions in cancer.

In lung cancer, LINC00261 is epigenetically regulated, with high methylation levels at the cg15058464 and cg07003030 loci, resulting in activation of the DNA damage response 16. In contrast, we demonstrated in our study that LINC00261 exhibits a high methylation level at the cg12179011 locus in PC and that this high methylation level is associated with poorer survival. These results suggest that methylation of LINC00261 can vary across tissues and organs. Because the benefits of chemotherapy are limited, patients with advanced PC have few treatment options. However, demethylation therapy 17, 41, 42, especially via the dCas9 system, which can target key tumor suppressor genes, may provide insight for curative therapies for PC. A novel technology reported in 2016 43, targeted demethylation therapy has already shown promise in treating other cancers. After the dCas9 system is constructed and transfected into cancer cells, it can precisely and specifically demethylate regions of the target gene, resulting in substantial activation of tumor suppressor gene expression. Our findings validated this therapy *in vitro* and *in vivo,* suggesting that targeted epigenetic therapies using dCas9-sgLINC00261 as a promising epigenetic drug target may be an alternate strategy for the treatment of PC, because this approach can restore the expression of LINC00261 and produce tumor inhibitory effects via c-Myc, ultimately improving the clinical prognosis of patients. Finally, regarding the methylation site cg12179011 that was our focus in the present study, our database prediction results indicated that the LINC00261 promoter contains multiple binding sites for several key transcription factors (**Figure S11B**). The methylation status at these sites might influence the functions of these transcription factors. Further studies are anticipated to reveal the related mechanisms. These collective results may help to circumvent the long-standing obstacles to PC treatment.

## Conclusion

These results indicate that LINC00261 acts as a novel potential tumor suppressor gene in PC through epigenetic inhibition of c-Myc transcription and subsequent suppression of c-Myc-driven PC cell proliferation, viability, migration and EMT-mediated invasion. Furthermore, downregulation of LINC00261 might occur due to the high methylation level of the LINC00261 promoter. Targeted demethylation of LINC00261 restores its expression and tumor inhibitory function. We anticipate that these findings will facilitate improvements in the current status of PC diagnosis and treatment.

## Supplementary Material

Supplementary figures, tables, and methods.

## Abbreviations

LncRNA: long noncoding RNA
PC: pancreatic cancer
TCGA: The Cancer Genome Atlas
GEO: Gene Expression Omnibus
c-Myc: v-myc avian myelocytomatosis viral oncogene homolog
MMP2: matrix metalloproteinase 2
MMP9: matrix metalloproteinase 9
CDK4: cyclin-dependent kinase 4
CCND1: cyclin D1
CDK6: cyclin-dependent kinase 6
HDAC1: histone deacetylase 1
HDAC2: histone deacetylase 2
VEGFA: vascular endothelial growth factor A
NC: normal control
ChIP: chromatin immunoprecipitation
RIP: RNA-binding protein immunoprecipitation
WB: western blot
FISH: fluorescence in situ hybridization
ISH: in situ hybridization
qRT-PCR: quantitative real-time polymerase chain reaction
PC: pancreatic cancer
NP: normal pancreas
ANT: adjacent nontumor tissue
DAPI: 4',6-diamidino-2-phenylindole dihydrochloride
DIG: digoxigenin
CY3: cyanine-3
P300: E1A binding protein p300
CBP: CREB binding protein
