# Supplementary material for: Methylation-mediated LINC00261 suppresses pancreatic cancer progression by epigenetically inhibiting c-Myc transcription
Source: Theranostics. 2020 Aug 25;10(23):10634–51. doi: 10.7150/thno.44278 (PMC7482811; doi:10.7150/thno.44278)
Supplement: Supplementary file 1 — Supplementary figures, tables, and methods. [file thnov10p10634s1.pdf]

Supplementary materials for

Methylation-mediated LINC00261 suppresses pancreatic cancer progression by epigenetically inhibiting c-Myc transcription

Authors: Songsong Liu[1], Yao Zheng[2], Yujun Zhang[1], Junfeng Zhang[2], Fuming Xie[2], Shixiang Guo[2], Jianyou Gu[3], Jiali Yang[1], Ping Zheng[1], Jiejuan Lai[1], Liangyu Yin[1,4*], Huaizhi Wang[1,2*]

Affiliations:

[1]Institute of Hepatopancreatobiliary Surgery, Southwest Hospital, Third Military Medical University (Army Medical University), Chongqing, P. R. China

[2]Institute of Hepatopancreatobiliary Surgery, Chongqing General Hospital, University of Chinese Academy of Sciences, Chongqing, P. R. China

[3]Department of First Hepatobiliary Surgery, Zhujiang Hospital, Southern Medical University, Guangzhou, Guangdong, P. R. China

[4]Department of Clinical Nutrition, Daping Hospital, Third Military Medical University (Army Medical University), Chongqing, P. R. China

***Corresponding authors:**

Huaizhi Wang

Institute of Hepatopancreatobiliary Surgery, Southwest Hospital, Third Military Medical University (Army Medical University), Chongqing, China

Institute of Hepatopancreatobiliary Surgery, Chongqing General Hospital, University of Chinese Academy of Sciences, Chongqing, China

E-mail: whuaizhi@gmail.com

Liangyu Yin

Institute of Hepatopancreatobiliary Surgery, Southwest Hospital, Third Military Medical University (Army Medical University), Chongqing, China

Department of Clinical Nutrition, Daping Hospital, Third Military Medical University (Army Medical University), Chongqing, China

E-mail: liangyuyin1988@gmail.com

Legend:

Figure S1. The workflow of the clinical samples used in the study.

Figure S2. PC and NP gene microarray information. **(A)** Hierarchical cluster analysis of differentially expressed genes in PC and normal pancreas (NP) tissues (fold change: top 2000; P < 0.05), GSE15471 (NP, N=39; PC, N=39), and GSE16515 (NP, N=16; PC, N=36). **(B)** Venn diagram of upregulated and downregulated genes in the two GEO datasets. (C) Kaplan-Meier analysis of survival of PC patients based on GEPIA (TCGA) database (PC, N=178). (D) The annotation of downregulated lncRNAs in GEPIA.

Figure S3. **(A)** LINC00261 expression in four PC cell lines, as analyzed by qRT-PCR. **(B)** qRT-PCR analysis of PANC-1 cells with siRNA-mediated LINC00261 knockdown and control PANC-1 cells. **(C)** qRT-PCR analysis of SW1990 cells with siRNA-mediated LINC00261 knockdown and control SW1990 cells. **(D)** qRT-PCR analysis of CFPAC-1 cells overexpressing LINC00261 via transfection of the pcDNA3.1 vector and control CFPAC-1 cells. **(E)** qRT-PCR analysis of BXPC-3 cells overexpressing LINC00261 via transfection of the pcDNA3.1 vector and control BXPC-3 cells.

Figure S4. LINC00261 significantly inhibited PC cell proliferation, migration and invasion *in vitro*. **(A, B)** EdU assays were used to assess the cell proliferation ability. Histogram showing the proliferation rates of transfected cells of corresponding groups. **(C)** CCK8 assays were used to assess the viability of transfected PC cells. **(D, E, F)** Transwell and Matrigel assays were used to assess transfected PC cells migration and invasion abilities. Histogram showing the number of migrated and invaded transfected PC cells. **(G)** WB analysis was performed to assess the expression of cell cycle-related markers. **(H)** WB analysis was performed to assess the expression of EMT-related markers (*P < 0.05, **P < 0.01, ***P < 0.001).

Figure S5. (A) E2F1, E2F7 and E2F8 gene expression levels were analyzed by qPCR in PANC-1 cells with LINC00261 knockdown and CFPAC-1 cells with LINC00261 overexpression. **(B)** Correlation analysis between LINC00261 and c-Myc-related

downstream molecules in TCGA. **(C)** Kaplan-Meier survival analysis of the four groups of PC patients based on TCGA data for LINC00261 and c-Myc expression levels. **(D)** c-Myc gene expression levels were analyzed by qPCR in SW1990 cells with LINC00261 knockdown and BXPC-3 cells with LINC00261 overexpression. **(E)** c-Myc gene expression levels were analyzed by WB in SW1990 cells with LINC00261 knockdown and BXPC-3 cells with LINC00261 overexpression. (ns: no significance, *P < 0.05, **P < 0.01, ***P < 0.001).

Figure S6. LINC00261 functions via the transcription factor c-Myc in PC**. (A-D)** c-Myc was downregulated using an inhibitor in cells with low LINC00261 expression. EdU, Transwell and Matrigel assays were used to assess proliferation, migration and invasion in PANC-1/SW1990 cells with NC, si-LINC00261 and siLINC00261+c-Myc-inhibitor (10058-F4) three groups. **(E)** Histogram showing the proliferation rates of PANC-1/SW1990 cells in the three groups. **(F and G)** Histogram showing the numbers of migrated and invaded PANC-1/SW1990 cells in the three groups. **(H and I)** mRNA levels of c-Myc and its related downstream molecules, as assessed by qRT-PCR, in PANC-1/SW1990 cells in the three groups. **(J)** Protein levels of c-Myc and its related downstream molecules, as assessed by WB analysis, in PANC-1/SW1990 cells in the three groups (*P < 0.05, **P < 0.01, ***P < 0.001).

Figure S7. (A) The protein levels of c-Myc and its related downstream molecules, as assessed by WB analysis, in SW1990 cells in the NC, si-LINC00261 and si-LINC00261+si-c-Myc groups; The protein levels of c-Myc and its related downstream molecules, as assessed by WB analysis, in BXPC-3 cells in the NC, ex-LINC00261 and ex-LINC00261+ex-c-Myc groups. **(B)** The mRNA levels of c-Myc and its related downstream molecules, as assessed by qRT-PCR, in SW1990 cells in the NC, si-LINC00261 and si-LINC00261+si-c-Myc groups. **(C)** The mRNA levels of c-Myc and its related downstream molecules, as assessed by qRT-PCR, in BXPC-3 cells the NC, ex-LINC00261 and ex-LINC00261+ex-c-Myc groups. **(D)** Luciferase activity assays were performed on LINC00261 knockdown PANC-1 and LINC00261-overexpressing CFPAC-1 cells cotransfected with the pGL3 reporter

vector containing the WT c-Myc promoter (promoter sequence starting 1000 bp upstream of the transcription start site). **(E)** The probabilities of LINC00261 interaction with its potential interacting proteins were predicted and calculated via an online database (http://pridb.gdcb.iastate.edu/RPISeq/) (RF or SVM scores > 0.5 are considered positive). **(F and H)** EdU, Transwell and Matrigel assays were used to assess proliferation, migration and invasion in the three groups of PANC-1 cells: si-NC, si-LINC00261 and si-LINC00261+ si-p300/CBP. **(G and I)** The EdU, Transwell and Matrigel assays were used to assess proliferation, migration and invasion in the three groups of CFPAC-1 cells: ex-NC, ex-LINC00261 and ex-LINC00261+ex-p300/CBP. (*P < 0.05, **P < 0.01, ***P < 0.001).

Figure S8. (A) The mRNA levels of p300/CBP was assessed by qRT-PCR, in the NC WT and mut-p300/CBP groups. **(B)**The WB levels of p300/CBP was assessed by WB analysis, in the WT and mut-p300/CBP groups. All plasmids were labeled with 3-flag. (*P < 0.05, **P < 0.01, ***P < 0.001).

Figure S9. (A) The diagram of sgRNAs targeting the cg1279011 site. The CpG sites are indicated in blue. **(B)** Assessment of the off-target effects of the dCas9-based demethylation system. The top 15 potential off-target sites predicted for LINC00261 were selected using a previously described scoring system. **(C)** qRT-PCR analysis of off-target mRNA expression levels in CFPAC-1/BXPC-3/PANC-1 cells transfected with EF1a-Dcas9-Tet1CD-CMV-EGFP/sgLINC00261 and sgRNA control (none: no expression in PC cells). (*P < 0.05, **P < 0.01, ***P < 0.001).

Figure S10. (A) The interaction profile, which represents the protein interaction score (Y-axis) relative to the LINC00261 sequence (X-axis), provides information about the region most likely to be bound by the protein. **(B)** The interaction matrix, which shows a heatmap of the interacting regions in the c-Myc protein (Y-axis) and LINC00261 (X-axis). The red shading in the heatmap indicates the interaction score of a single amino acid and nucleotide pair. **(C)** RIP assays were performed to validate LINC00261 binding to c-Myc in PC cells. **(D)** c-Myc-overexpressing PC cells were transfected with ex-LINC00261 or ex-NC. Luciferase activity assays were performed to detect the activity of the CDK4 promoter reporter construct (CDK4 pGL3 reporter

vector; sequence beginning 781 bp upstream of the TSS). **(E and F)** c-Myc-overexpressing PC cells were transfected with ex-LINC00261 or ex-NC. The mRNA and protein levels of c-Myc in the NC, ex-c-Myc and ex-c-Myc+ex-LINC00261 groups were assessed by qRT-PCR or WB analysis. **(G)** Schematic model of the possible mechanism by which LINC00261 regulates CDK4 by competitively interacting with c-Myc (*P < 0.05, **P < 0.01, ***P < 0.001).

Figure S11. (A) IP assays were performed to validate the interaction of p300/CBP and related transcriptional factors in LINC00261 overexpression and knockdown PC cells. **(B)** Predicted transcription factors binding to the cg12179011 site (factors predicted by PROMO).

Table S1. Information about the primers used for qRT-PCR, RIP, and ChIP; siRNA sequences; antibodies used for WB and IHC; and probes used for FISH.

Table S2. Supplemental materials and methods.

Table S3. Baseline characteristics of the 205 study participants stratified by the LINC00261 expression level.

Table S4. Univariate and multivariate Cox proportional hazards regression analyses of the association between the LINC00261 expression level and survival.

Figure S1

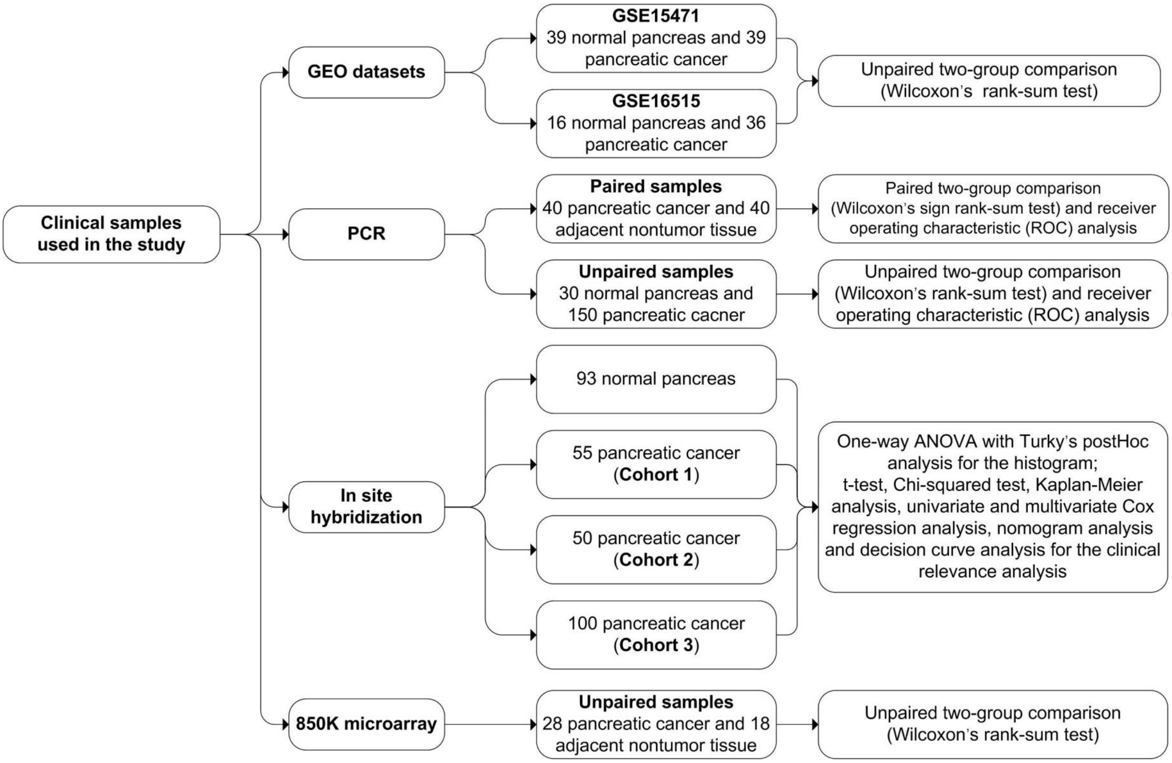

Clinical samples used in the study
GEO datasets
GSE15471
39 normal pancreas and 39 pancreatic cancer
GSE16515
16 normal pancreas and 36 pancreatic cancer
Unpaired two-group comparison (Wilcoxon's rank-sum test)
PCR
Paired samples
40 pancreatic cancer and 40 adjacent nontumor tissue
Paired two-group comparison (Wilcoxon's sign rank-sum test) and receiver operating characteristic (ROC) analysis
Unpaired samples
30 normal pancreas and 150 pancreatic cacner
Unpaired two-group comparison (Wilcoxon's rank-sum test) and receiver operating characteristic (ROC) analysis
In site hybridization
93 normal pancreas
55 pancreatic cancer (Cohort 1)
50 pancreatic cancer (Cohort 2)
100 pancreatic cancer (Cohort 3)
One-way ANOVA with Turky's postHoc analysis for the histogram; t-test, Chi-squared test, Kaplan-Meier analysis, univariate and multivariate Cox regression analysis, nomogram analysis and decision curve analysis for the clinical relevance analysis
850K microarray
Unpaired samples
28 pancreatic cancer and 18 adjacent nontumor tissue
Unpaired two-group comparison (Wilcoxon's rank-sum test)

Figure S2

A

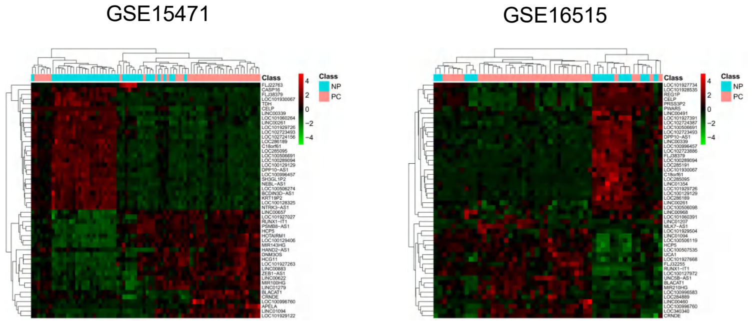

B

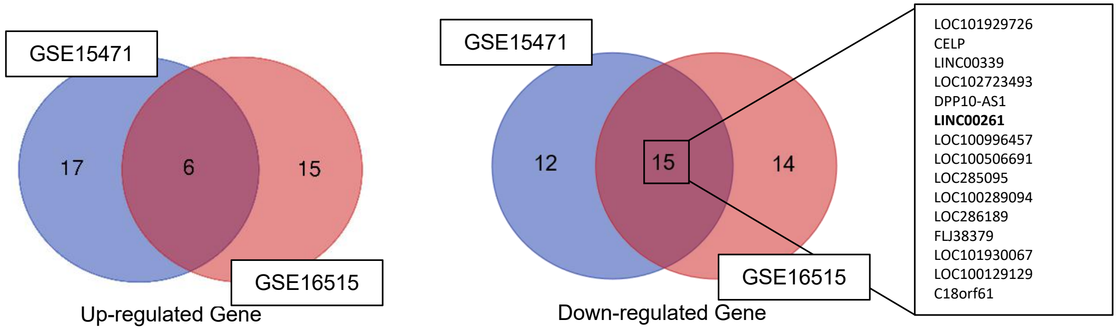

C

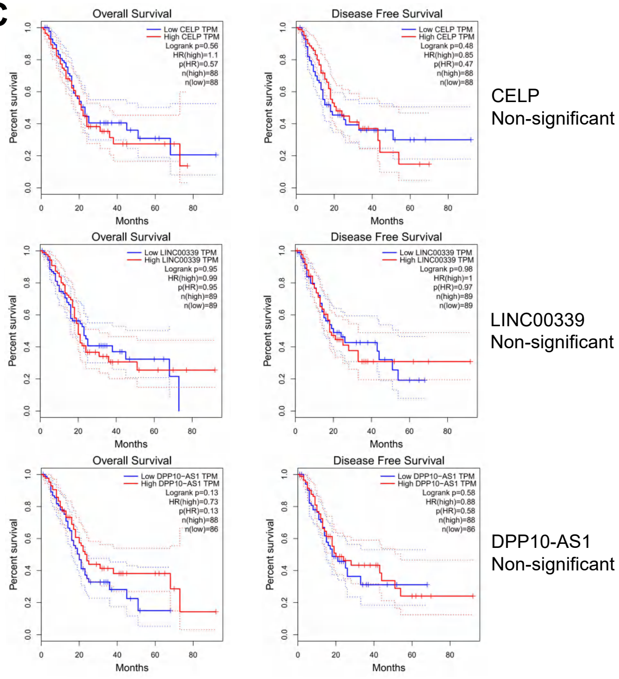

D

Symbol	Annotation
LOC101929726	None
CELP25	Annotatied
LINC00339	Annotatied
LOC102723493	None
DPP10-AS1	Annotatied
**LINC00261**	Annotatied
LOC100996457	None
LOC100506691	None
LOC285095	None
LOC100289094	None
LOC286189	None
FLJ38379	None
LOC101930067	None
LOC100129129	None
C18orf61	None

Figure S3

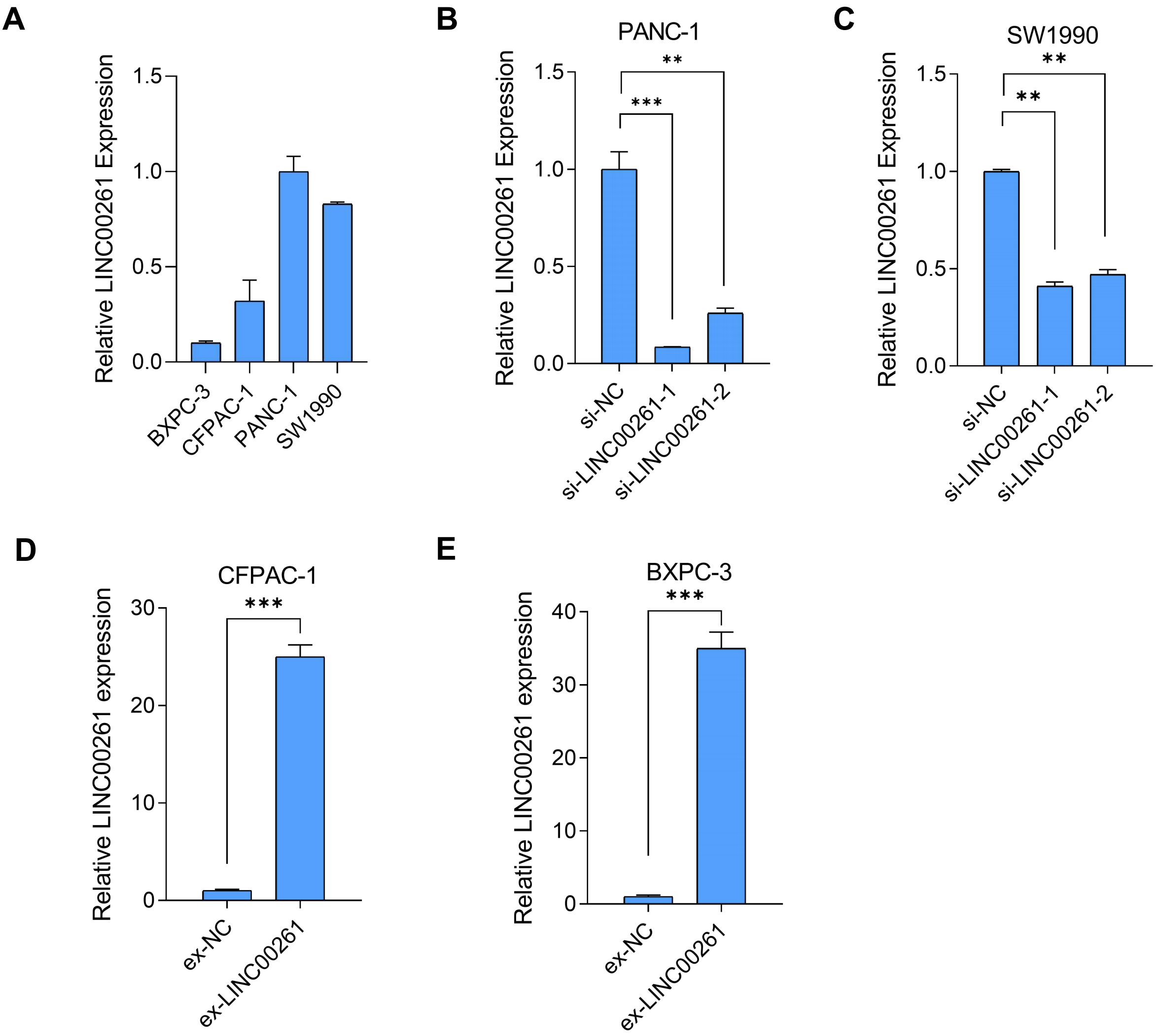
A
Relative LINC00261 Expression
1.5
1.0
0.5
0.0
BXPC-3
CFPAC-1
PANC-1
SW1990
B
PANC-1
**
***
Relative LINC00261 Expression
si-NC
si-LINC00261-1
si-LINC00261-2
C
SW1990
**
**
Relative LINC00261 Expression
si-NC
si-LINC00261-1
si-LINC00261-2
D
CFPAC-1
***
Relative LINC00261 expression
30
20
10
0
ex-NC
ex-LINC00261
E
BXPC-3
***
Relative LINC00261 expression
40
30
20
10
0
ex-NC
ex-LINC00261

Figure S4

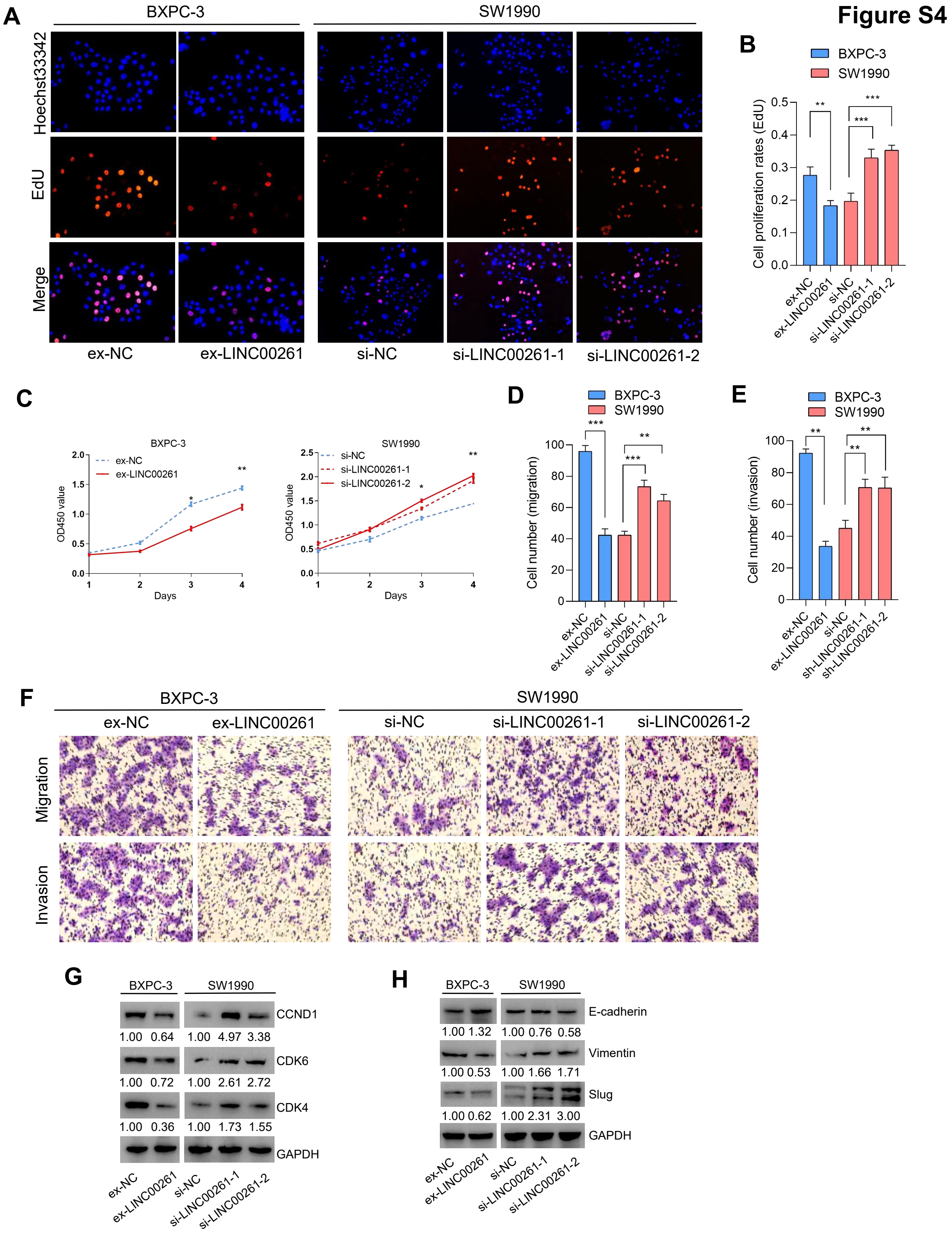
A
BXPC-3
SW1990
Hoechst33342
EdU
Merge
ex-NC
ex-LINC00261
si-NC
si-LINC00261-1
si-LINC00261-2
B
BXPC-3
SW1990
Cell proliferation rates (EdU)
0.0
0.1
0.2
0.3
0.4
0.5
**
***
***
ex-NC
ex-LINC00261
si-NC
si-LINC00261-1
si-LINC00261-2
C
BXPC-3
ex-NC
ex-LINC00261
OD450 value
Days
*
**
SW1990
si-NC
si-LINC00261-1
si-LINC00261-2
D
BXPC-3
SW1990
Cell number (migration)
***
***
**
ex-NC
ex-LINC00261
si-NC
si-LINC00261-1
si-LINC00261-2
E
BXPC-3
SW1990
Cell number (invasion)
**
**
**
ex-NC
ex-LINC00261
si-NC
sh-LINC00261-1
sh-LINC00261-2
F
BXPC-3
SW1990
ex-NC
ex-LINC00261
si-NC
si-LINC00261-1
si-LINC00261-2
Migration
Invasion
G
BXPC-3
SW1990
CCND1
1.00 0.64 1.00 4.97 3.38
CDK6
1.00 0.72 1.00 2.61 2.72
CDK4
1.00 0.36 1.00 1.73 1.55
GAPDH
ex-NC
ex-LINC00261
si-NC
si-LINC00261-1
si-LINC00261-2
H
BXPC-3
SW1990
E-cadherin
1.00 1.32 1.00 0.76 0.58
Vimentin
1.00 0.53 1.00 1.66 1.71
Slug
1.00 0.62 1.00 2.31 3.00
GAPDH
ex-NC
ex-LINC00261
si-NC
si-LINC00261-1
si-LINC00261-2

Figure S5

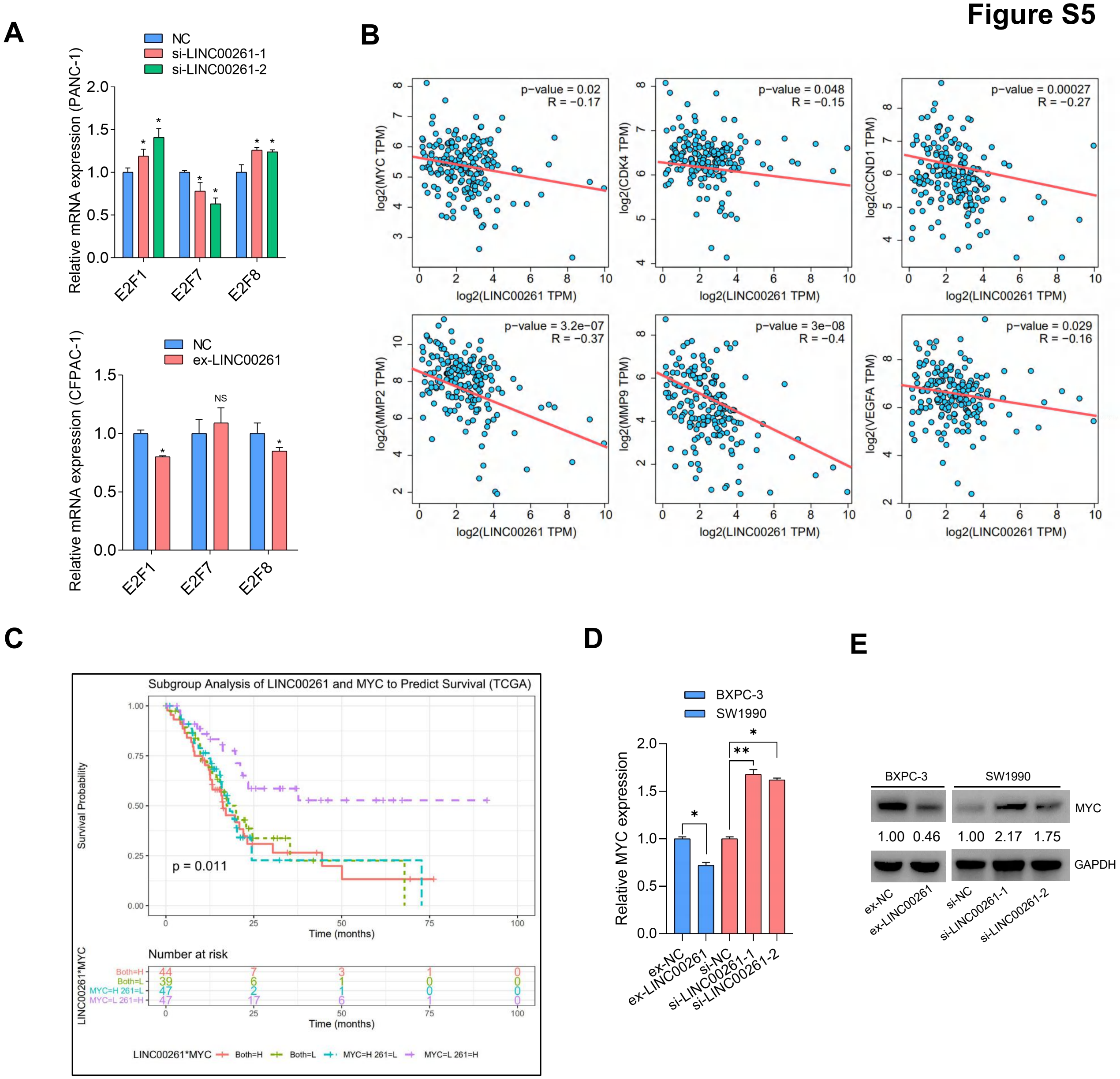

A
NC
si-LINC00261-1
si-LINC00261-2
Relative mRNA expression (PANC-1)
E2F1
E2F7
E2F8
NC
ex-LINC00261
Relative mRNA expression (CFPAC-1)
NS
B
p−value = 0.02
R = −0.17
log2(MYC TPM)
p−value = 0.048
R = −0.15
log2(CDK4 TPM)
p−value = 0.00027
R = −0.27
log2(CCND1 TPM)
p−value = 3.2e−07
R = −0.37
log2(MMP2 TPM)
p−value = 3e−08
R = −0.4
log2(MMP9 TPM)
p−value = 0.029
R = −0.16
log2(VEGFA TPM)
log2(LINC00261 TPM)
C
Subgroup Analysis of LINC00261 and MYC to Predict Survival (TCGA)
Survival Probability
p = 0.011
Time (months)
Number at risk
LINC00261*MYC
Both=H 44 7 3 1 0
Both=L 39 6 1 0 0
MYC=H 261=L 47 2 1 0 0
MYC=L 261=H 47 17 6 1 0
LINC00261*MYC Both=H Both=L MYC=H 261=L MYC=L 261=H
D
BXPC-3
SW1990
Relative MYC expression
ex-NC
ex-LINC00261
si-NC
si-LINC00261-1
si-LINC00261-2
E
BXPC-3
SW1990
MYC
1.00 0.46 1.00 2.17 1.75
GAPDH
ex-NC
ex-LINC00261
si-NC
si-LINC00261-1
si-LINC00261-2

Figure S6

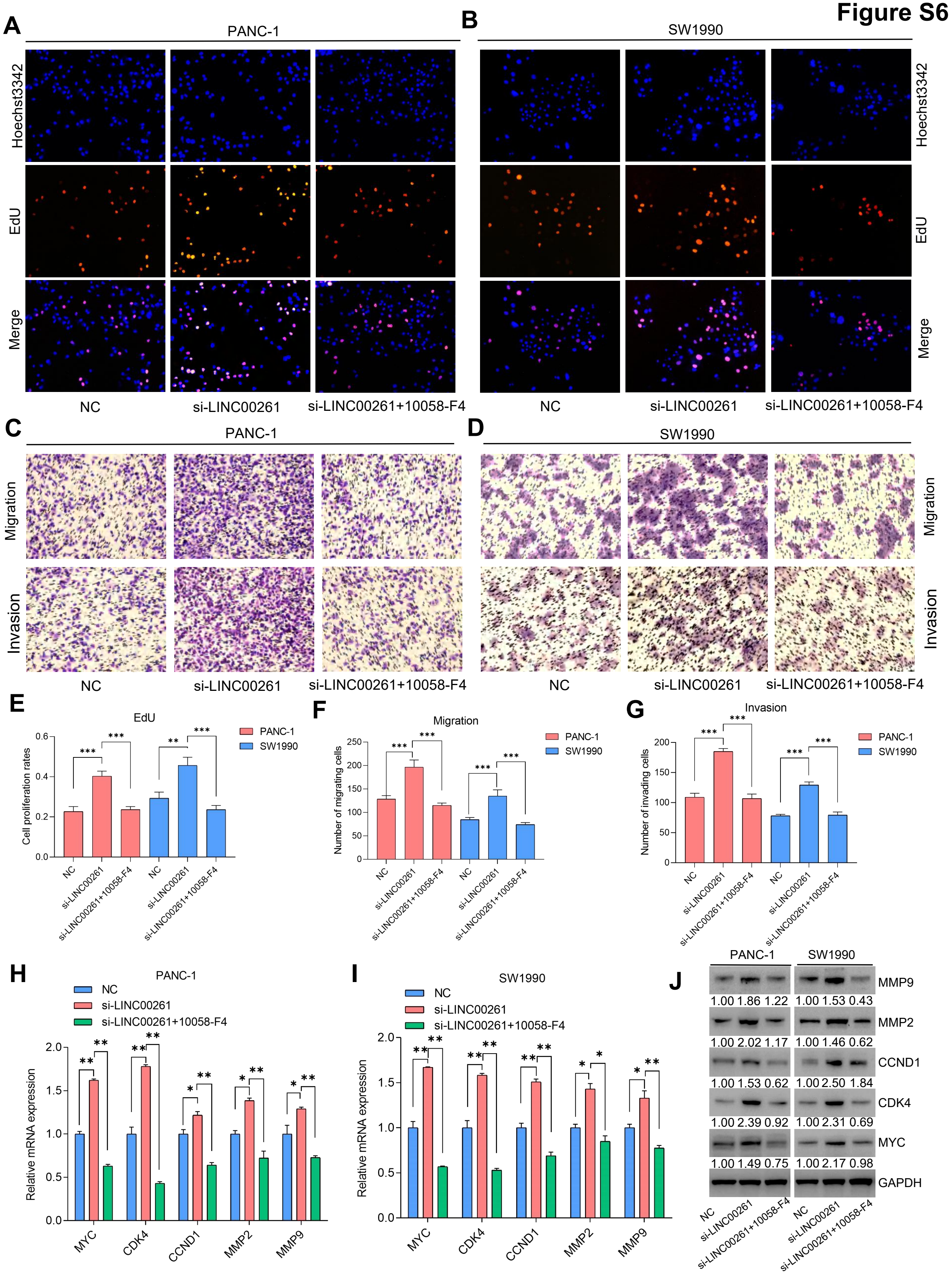

A
PANC-1
Hoechst3342
EdU
Merge
NC
si-LINC00261
si-LINC00261+10058-F4
B
SW1990
Hoechst3342
EdU
Merge
NC
si-LINC00261
si-LINC00261+10058-F4
C
PANC-1
Migration
Invasion
NC
si-LINC00261
si-LINC00261+10058-F4
D
SW1990
Migration
Invasion
NC
si-LINC00261
si-LINC00261+10058-F4
E
EdU
Cell proliferation rates
PANC-1
SW1990
F
Migration
Number of migrating cells
PANC-1
SW1990
G
Invasion
Number of invading cells
PANC-1
SW1990
H
PANC-1
NC
si-LINC00261
si-LINC00261+10058-F4
Relative mRNA expression
MYC
CDK4
CCND1
MMP2
MMP9
I
SW1990
NC
si-LINC00261
si-LINC00261+10058-F4
Relative mRNA expression
MYC
CDK4
CCND1
MMP2
MMP9
J
PANC-1
SW1990
MMP9
1.00 1.86 1.22 1.00 1.53 0.43
MMP2
1.00 2.02 1.17 1.00 1.46 0.62
CCND1
1.00 1.53 0.62 1.00 2.50 1.84
CDK4
1.00 2.39 0.92 1.00 2.31 0.69
MYC
1.00 1.49 0.75 1.00 2.17 0.98
GAPDH
NC
si-LINC00261
si-LINC00261+10058-F4

Figure S7

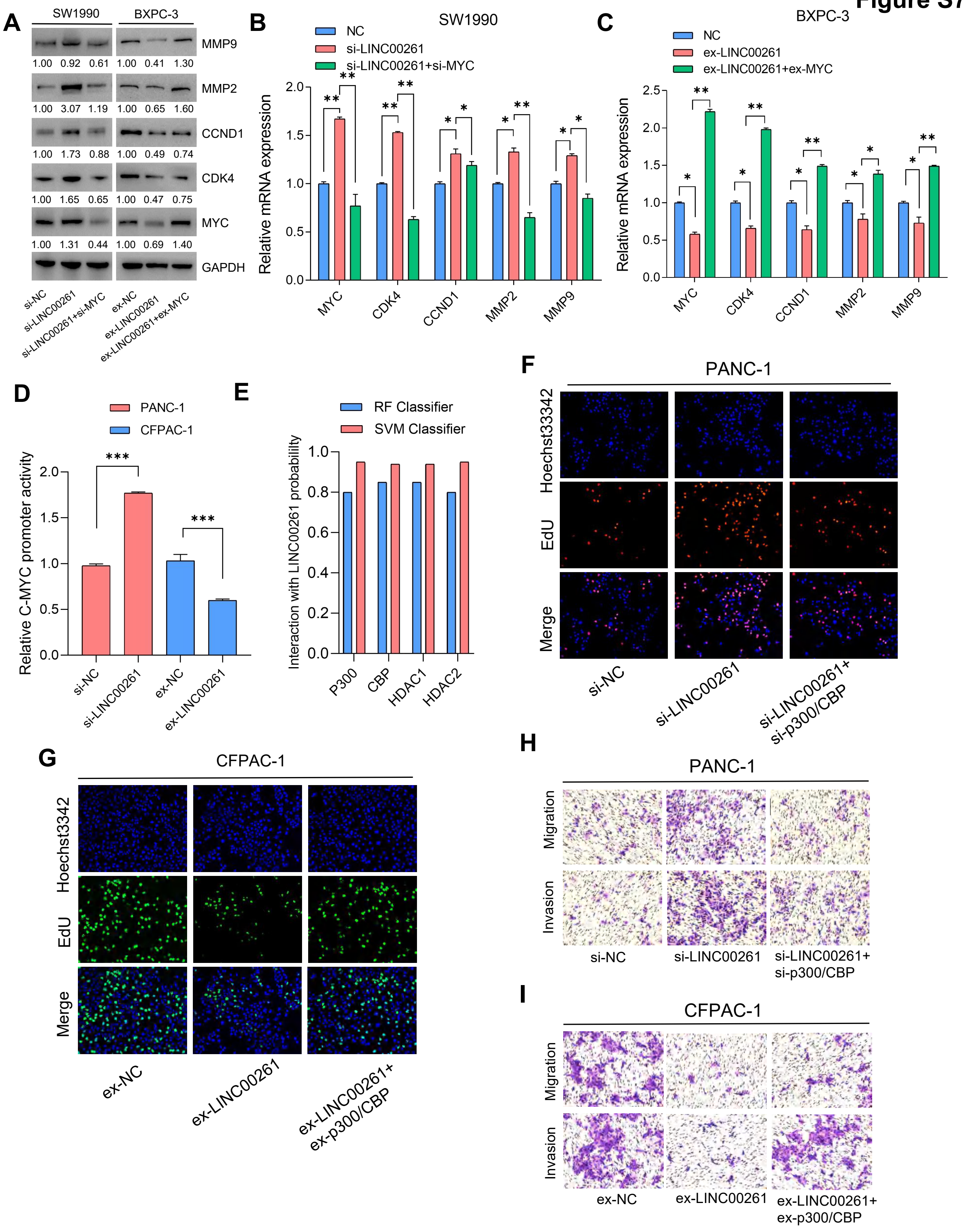
A
SW1990
BXPC-3
MMP9
1.00 0.92 0.61 1.00 0.41 1.30
MMP2
1.00 3.07 1.19 1.00 0.65 1.60
CCND1
1.00 1.73 0.88 1.00 0.49 0.74
CDK4
1.00 1.65 0.65 1.00 0.47 0.75
MYC
1.00 1.31 0.44 1.00 0.69 1.40
GAPDH
si-NC
si-LINC00261
si-LINC00261+si-MYC
ex-NC
ex-LINC00261
ex-LINC00261+ex-MYC
B
SW1990
NC
si-LINC00261
si-LINC00261+si-MYC
Relative mRNA expression
MYC
CDK4
CCND1
MMP2
MMP9
C
BXPC-3
NC
ex-LINC00261
ex-LINC00261+ex-MYC
Relative mRNA expression
MYC
CDK4
CCND1
MMP2
MMP9
D
PANC-1
CFPAC-1
Relative C-MYC promoter activity
si-NC
si-LINC00261
ex-NC
ex-LINC00261
E
RF Classifier
SVM Classifier
Interaction with LINC00261 probability
P300
CBP
HDAC1
HDAC2
F
PANC-1
Hoechst33342
EdU
Merge
si-NC
si-LINC00261
si-LINC00261+
si-p300/CBP
G
CFPAC-1
Hoechst33342
EdU
Merge
ex-NC
ex-LINC00261
ex-LINC00261+
ex-p300/CBP
H
PANC-1
Migration
Invasion
si-NC
si-LINC00261
si-LINC00261+
si-p300/CBP
I
CFPAC-1
Migration
Invasion
ex-NC
ex-LINC00261
ex-LINC00261+
ex-p300/CBP

Figure S8

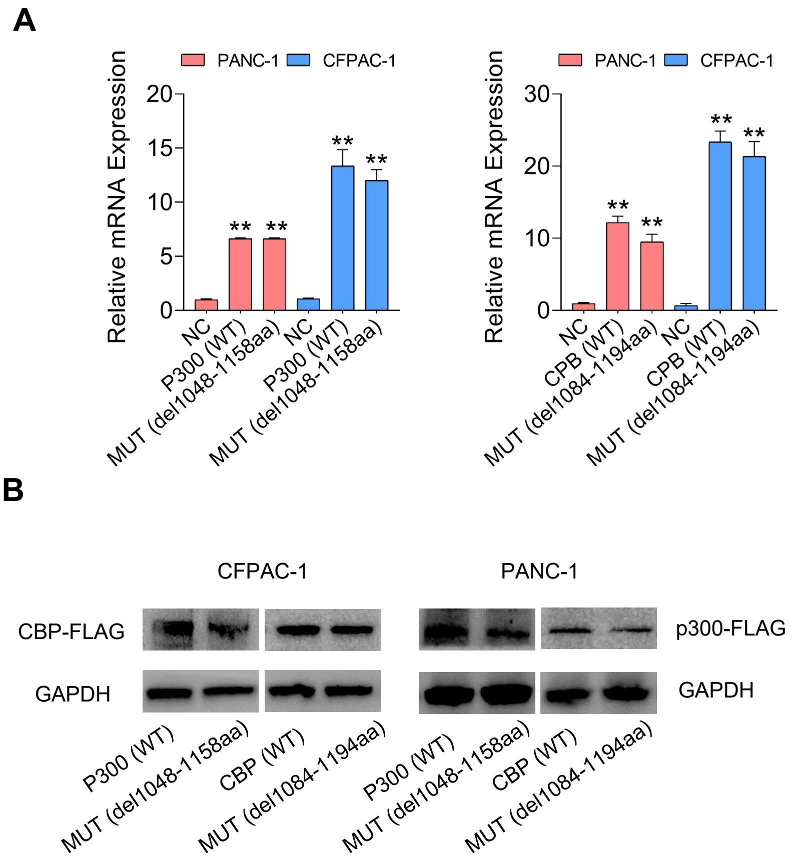

A
PANC-1
CFPAC-1
Relative mRNA Expression
20
15
10
5
0
**
NC
P300 (WT)
MUT (del1048-1158aa)
NC
P300 (WT)
MUT (del1048-1158aa)
30
20
10
0
NC
CPB (WT)
MUT (del1084-1194aa)
NC
CPB (WT)
MUT (del1084-1194aa)
B
CFPAC-1
PANC-1
CBP-FLAG
p300-FLAG
GAPDH
GAPDH
P300 (WT)
MUT (del1048-1158aa)
CBP (WT)
MUT (del1084-1194aa)
P300 (WT)
MUT (del1048-1158aa)
CBP (WT)
MUT (del1084-1194aa)

Figure S9

A

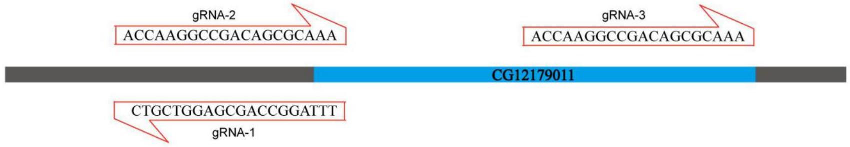

B

Gene	Chromosome	Strand	Target sequence	Mismatches	Score
VIT	2	+	ATTAGGCCAGAGAGGACTTCCGG	4	0.45
ZNF500	16	+	TTAAAGCCAGCAAAGTCGTCCGG	4	0.43
PYHIN1	1	+	TTTAGGACATCAAGGTCTTCAGG	4	0.38
LSM3	3	+	TTTTGGCCAGCAGGGCCGTCTGG	4	0.26
CACHD1	1	+	ATTAGGCCAGAGAGGCAGTCGGG	4	0.23
LINC00701	10	+	ACAAGGGCTGACAGCACAAATGG	4	0.55
CDH4	20	+	ACAAAGGAAGACAGCACAAAAGG	4	0.48
ANKRD44	2	+	ACCAAGGATGGCAGCACAAAAGG	4	0.37
SDK1	7	+	ACCAAGTCCAGCAGCACAAAAGG	4	0.35
AC017002	2	-	ACCAAGGCTGAGAGCACAAGAGG	4	0.30
ENOX1	13	-	GCCCAGAACATCAGCTGGCTTGG	2	0.69
PISD	22	-	GCACAGGAGATCTGCTGGCTCGG	2	0.54
LSINCT5	5	-	AACAGGGACATCTGCTGGCTGGG	4	0.40
ITPKB	1	-	ACCCAGCACATCCACTGGCTAGG	4	0.37
NTRK3	15	-	CCCCAAGACATCTGCAGGCAGGG	4	0.37

C

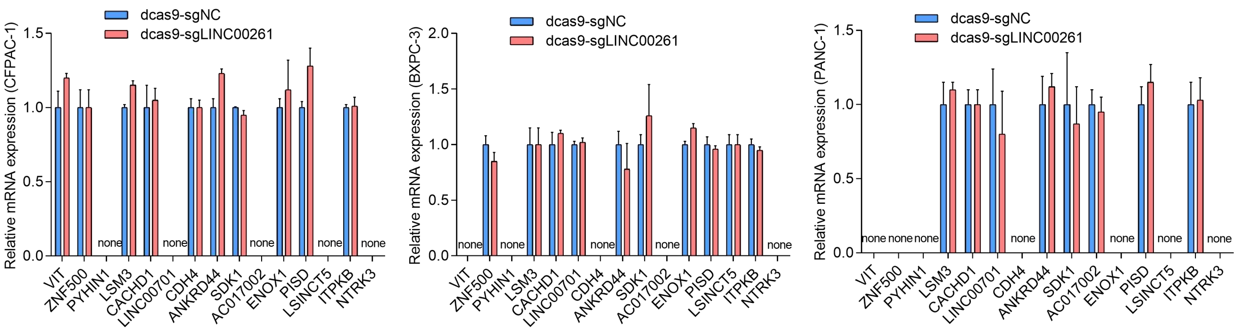

Figure S10

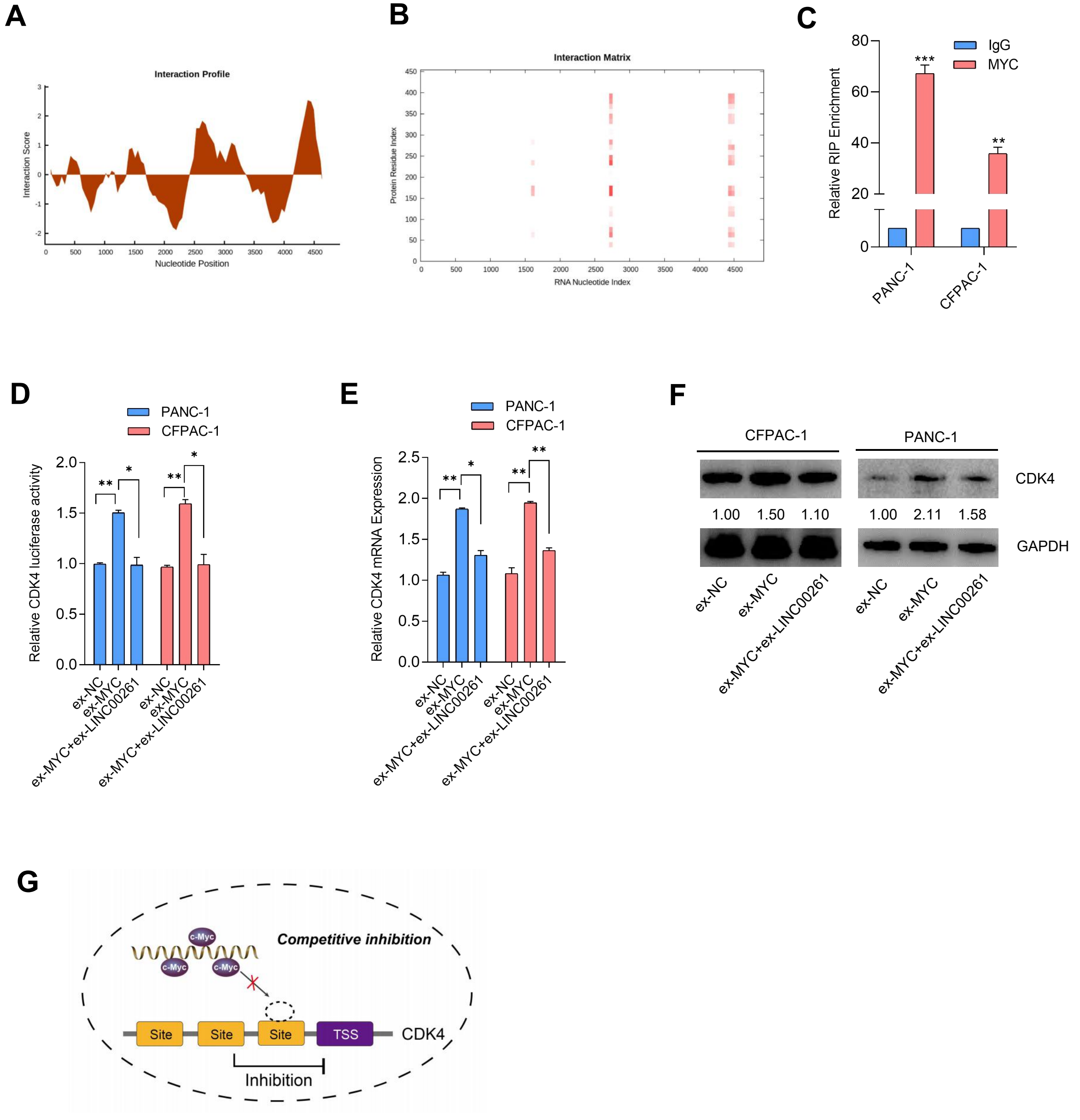
A
Interaction Profile
Interaction Score
Nucleotide Position
B
Interaction Matrix
Protein Residue Index
RNA Nucleotide Index
C
IgG
MYC
Relative RIP Enrichment
***
**
PANC-1
CFPAC-1
D
PANC-1
CFPAC-1
Relative CDK4 luciferase activity
**
*
ex-NC
ex-MYC
ex-MYC+ex-LINC00261
E
PANC-1
CFPAC-1
Relative CDK4 mRNA Expression
**
*
ex-NC
ex-MYC
ex-MYC+ex-LINC00261
F
CFPAC-1
PANC-1
CDK4
1.00 1.50 1.10
1.00 2.11 1.58
GAPDH
ex-NC
ex-MYC
ex-MYC+ex-LINC00261
G
c-Myc
Competitive inhibition
Site
Site
Site
TSS
CDK4
Inhibition

Figure S11

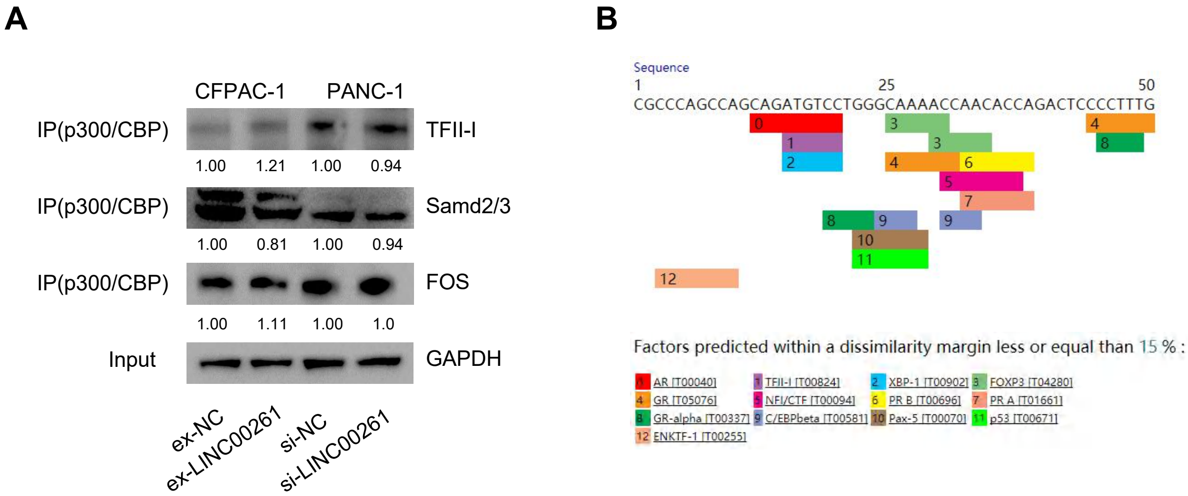
A
CFPAC-1
PANC-1
IP(p300/CBP)
TFII-I
1.00 1.21 1.00 0.94
IP(p300/CBP)
Samd2/3
1.00 0.81 1.00 0.94
IP(p300/CBP)
FOS
1.00 1.11 1.00 1.0
Input
GAPDH
ex-NC
ex-LINC00261
si-NC
si-LINC00261
B
Sequence
1
25
50
CGCCCAGCCAGCAGATGTCCTGGGCAAAACCAACACCAGACTCCCCTTTG
Factors predicted within a dissimilarity margin less or equal than 15 % :
0 AR [T00040]
1 TFII-I [T00824]
2 XBP-1 [T00902]
3 FOXP3 [T04280]
4 GR [T05076]
5 NFI/CTF [T00094]
6 PR B [T00696]
7 PR A [T01661]
8 GR-alpha [T00337]
9 C/EBPbeta [T00581]
10 Pax-5 [T00070]
11 p53 [T00671]
12 ENKTF-1 [T00255]

Table S1

**Information of the primer.**	
**qRT-PCR primer name**	**primer sequence (5'-3')**
GAPDH (Forward)	CAGGAGGCATTGCTGATGAT
GAPDH (Reverse)	GAAGGCTGGGGCTCATTT
β-actin (Forward)	CCTGGCACCCAGCACAAT
β-actin (Reverse)	GGGCCGGACTCGTCATAC
c-MYC (Forward)	GTGGCACCTCTTGAGGACC
c-MYC (Reverse)	TGGTGCTCCATGAGGAGACA
CDK4 (Forward)	GTGTATGGGGCCGTAGGAAC
CDK4 (Reverse)	CAGTCGCCTCAGTAAAGCCA
CCND1(Forward)	GATGCCAACCTCCTCAACGA
CCND1(Reverse)	GGAAGCGGTCCAGGTAGTTC
VEGFA (Forward)	AAAACACAGACTCGCGTTGC
VEGFA (Reverse)	GTCGATGGTGATGGTGTGGT
LINC00261 (Forward)	CACAGCACCCTCAACAATGC
LINC00261 (Reverse)	AGCTCTCTCCCCATTAGCCA
MMP2 (Forward)	GTGGATGATGCCTTTGCTCG
MMP2 (Reverse)	GGAGTCCGTCCTTACCGTCAA
MMP9 (Forward)	CCCTTGTGCTCTTCCCTGGA
MMP9 (Reverse)	TCTGCCACCCGAGTGTAACC
E2F1 (Forward)	CACTTTCGGCCCTTTTGCTC
E2F1 (Reverse)	GATTCCCCAGGCTCACCAAA
E2F7 (Forward)	GCAGTGGTTGTTTCTGTCAGG
E2F7 (Reverse)	AACCCTGGTCAGTGTAGGGC
E2F8 (Forward)	GGACAGTACCTGCTTGCCTT
E2F8 (Reverse)	GGAGGTCGCTTGACAGGAAA
p300 (Forward)	CAGGGCCTAACATGGGACAG
p300 (Reverse)	CGAGGCATCATCTGGTTTGG
CBP (Forward)	GAGAACTTGCTGGACGGACC
CBP (Reverse)	GCTGTCATTCGCCGAGAAAC
**RIP qRT-PCR primer name**	**primer sequence (5'-3')**
LINC00261 (Forward)	CACAGCACCCTCAACAATGC
LINC00261 (Reverse)	AGCTCTCTCCCCATTAGCCA
**CHIP qRT-PCR primer name**	**primer sequence (5'-3')**
c-MYC (Forward)	TCATAACGCGCTCTCCAAGT
c-MYC (Reverse)	CTCGCTAAGGCTGGGGAAAG
c-MYC H3K27Ac (Forward)	TCATAACGCGCTCTCCAAGT
c-MYC H3K27Ac (Reverse)	CTCGCTAAGGCTGGGGAAAG
**pyrosequencing analysis**	

cg12179011-F	GGTGTTGGTTTTGTTTAGGATATT
cg12179011-Rbio	ATAATCAAAAAACCCAAAAATCTAATCT
cg12179011-S	GGATATTTGTTGGTTGG
**Off-target qRT-PCR primer name**	
VIT (Forward)	AGGTTCTTGGACCTCTCCCT
VIT (Reverse)	TGAGGCACAGTGAACTTGGG
ZNF500 (Forward)	GCCCTGTGGAGAGACGATAAA
ZNF500 (Reverse)	TCTGTCAGCAACCTGTGTGT
PYHIN1 (Forward)	GTGGCCCCTCCTCTTTCTTC
PYHIN1 (Reverse)	GGTGGAGGACTTCAGGCAAA
LSM3-1F (Forward)	AACCTGTTTACAGCAGCCCA
LSM3-1F (Reverse)	TACTAATGACCACAGGCGGC
CACHD1 (Forward)	GCCTTCAATCCAGGACGAGA
CACHD1 (Reverse)	CACAGAAATCTACTGCGGTGT
LINC00701 (Forward)	GCCTTCAATCCAGGACGAGA
LINC00701(Reverse)	CACAGAAATCTACTGCGGTGT
CDH4 (Forward)	AAGGCTGGGTTCTCTGAAGATG
CDH4 (Reverse)	ACGGCGTCCCATTTCTCTG
ANKRD44 (Forward)	ATTTGCCAGTGTCTGCCTGT
ANKRD44 (Reverse)	CACACCAAGTTGCTCAACAGT
SDK1 (Forward)	CAGGCCTACCACAGATCCAC
SDK1 (Reverse)	CCATTCTGTTTCGCACCACG
AC017002 (Forward)	CCCCCTAATGTTTGGTGCAGA
AC017002 (Reverse)	GCCCTATGGCCACTCATTCA
ENOX1 (Forward)	GCCTTCAATCCAGGACGAGA
ENOX1 (Reverse)	CACAGAAATCTACTGCGGTGT
PISD (Forward)	AGCCTCCATCCCTGTGAGAT
PISD (Reverse)	ACCAGCAAGTTTGGGTGGAA
LSINCT5 (Forward)	TCTCCTCCCCTCCAAACACA
LSINCT5 (Reverse)	CTTCCTTGCTTTCATGGGCG
ITPKB (Forward)	AACATGGTGCACTGGTCTCC
ITPKB (Reverse)	TCGTCCATCTGGTTGTAGCG
NTRK3 (Forward)	GCGTTTCAAAGAAGCAGCGA
NTRK3 (Reverse)	CTGAATCCTGCCCTTCCAGG

siRNA name	target sequence (5'-3')
siRNA-LINC00261-1	GAAAGCTGTAGCCATTCAA
siRNA-LINC00261-2	GCAATTAATTCAGGACACT
siRNA NC	TTCTCCGAACGTGTCACGT
siRNA c-MYC	GAGGAGACATGGTGAACCA
siRNA p300	CAGAGCAGTCCTGGATTAGTT
siRNA CBP	GGAGCCATCTAGTGCATAATT
**EF1a-Dcas9-Tet1CD-CMV-EGFP**	**target sequence (5'-3')**
sgRNA1	TTTAGGCCAGCGAGGTCGTC
sgRNA2	ACCAAGGCCGACAGCGCAAA
sgRNA3	GCCCAGGACATCTGCTGGCT
sgNC	CGCTTCCGCGGCCCGTTCAA

Antibody	band	application	number
MYC	CST	WB(1/1000)/CHIP/RIP(5ug)	2276
MYC	BIOSS	IHC(1/200)	bs-4963R
Ki67	Proteintech	IHC(1/100)	27309-1-AP
MMP2	Proteintech	WB(1/1000)	10373-2-AP
CCND1	Cell Signaling Technologies	WB(1/1000)	55506
MMP9	Proteintech	WB(1/1000)	10375-2-AP
CDK4	Cell Signaling Technologies	WB(1/1000)	12790
CDK6	Cell Signaling Technologies	WB(1/1000)	13331
GAPDH	Proteintech	WB(1/1000)	10494-1-AP
H3K27Ac	Cell Signaling Technologies	CHIP(5ug)	8173
E-CADHERIN	Proteintech	WB(1/1000)	20874-1-AP
N-CADHERIN	Proteintech	WB(1/1000)	22018-1-AP
VIMENTIN	Proteintech	WB(1/1000)	10366-1-AP
Slug	Proteintech	WB(1/1000)	12129-1-AP
HDAC1	Cell Signaling Technologies	RIP(5ug)	34589
HDAC2	Cell Signaling Technologies	RIP(5ug)	57156
p300	Cell Signaling Technologies	RIP/CHIP(5ug)	54062
CBP	Cell Signaling Technologies	RIP/CHIP(5ug)	7389
FLAG	Santa	RIP(5ug)/IP(2ug)	14793
SMAD2/3	Cell Signaling Technologies	WB(1/1000)	8685
TFII-I	Cell Signaling Technologies	WB(1/1000)	4562
**Inhibitors**			
10058-F4	Selleck	20uM	S7153

ISH/FISH PROBE	probe sequence (5'-3')
LINC00261	/5Dig/AATGTGTCACTGAATCTGCGT/3Dig/
LINC00261	/5CY3/AATGTGTCACTGAATCTGCGT/3CY3/

Table S2

Methods

Cohorts and tissue samples

All fresh-frozen tissue samples, including the PC tissues with adjacent noncancerous tissues used for qPCR (30 NP tissues and 150 PC tissues, 40 pairs of PC and adjacent nontumor tissues), were obtained from the Institute of Hepatopancreatobiliary Surgery, Southwest Hospital, Army Medical University. Of the 298 formalin-fixed, paraffin-embedded tissues contained in the two independent tissue microarrays, 55 PC (Cohort 1) and 13 NP tissues were obtained from the archival collections of Southwest Hospital, 50 PC tissues were obtained from Soochow University (Cohort 2) and 100 PC and 80 NP tissues were purchased from Outdo Biotech (Shanghai, China) (Cohort 3). For the 850k microarray, 28 PC tissues and 18 adjacent noncancerous tissues were obtained from the Southwest Hospital. NP samples were obtained from organ donors. The pathological type of all PC tissues in the present study was pancreatic ductal adenocarcinoma, and diagnoses were made based on surgical pathology. No patient received either chemotherapy or radiotherapy before surgery. The clinical stage was evaluated based on the guidelines in the American Joint Committee on Cancer (AJCC) 7th edition. Resected specimens were cut into blocks of a proper size, immediately submerged in the RNA preserving reagent RNAlater (Thermo, USA) and either frozen in liquid nitrogen for further RNA extraction and qPCR analysis or formaldehyde fixed and paraffin embedded for further histological analysis. Each sample was used for only one specific type of assay, for example, in situ hybridization (ISH) or qPCR. Follow-up was performed every three months after surgery to obtain the survival status. The study protocol was approved by the Ethics Committee of Southwest Hospital (No. KY201875), Army Medical University. All patients provided written informed consent upon admission for the use of human specimens. All procedures, including the use of human tissue specimens and analysis of clinical data, were carefully handled to meet the guidelines of the Declaration of Helsinki.

Microarray analysis

Two independent datasets, GSE15471 (including 39 NP and 39 PC tissues) and GSE16515 (including 16 NP and 36 PC tissues), were downloaded from the Gene Expression Omnibus (GEO). The original log^2 transformed normalized signaling data were processed, and differential analyses were performed using the R package limma (downloaded from www.bioconductor.org). The differentially expressed lncRNAs were visualized using the R package pheatmap. All bioinformatic analyses were performed in R (version 3.6.1, www.r-project.org). The intersection of the differentially expressed lncRNAs in both datasets was visualized via a Venn diagram generated using an online tool (bioinformatics.psb.ugent.be/webtools/Venn/). In addition, the Gene Expression Profiling Interactive Analysis (GEPIA, gepia.cancer-pku.cn) online database was used to estimate the associations between lncRNA expression levels and survival.

RNA sequencing analysis

PANC-1 cells were transfected with si-LINC00261 and control siRNA. Biological triplicates were established for each group. Total RNA was extracted from the samples using the TRIzol method. The quality and purity of total RNA extracted from the samples were analyzed by NanoDrop 2000 measurement and denaturing gel electrophoresis. RNA-seq was performed by SHBIO (Shanghai, China) with the Illumina HiSeq 2500 platform. Differential analyses between the si-LINC00261 group and the control group were performed using the R package limma. Detailed information about the RNA-seq protocol, including the sample preparation methods, study design and raw sequencing data, has been uploaded to the GEO database (GSE152012). All the original data would be scheduled to be released on Jun 8, 2023.

DNA methylation analysis

Genomic DNA was extracted from cells and tissues (OMEGA, USA). The screening of methylation levels in PC tissues and adjacent noncancerous tissues was performed using

MethylationEPIC BeadChip (Illumina, USA). For pyrosequencing analysis, genomic DNA was treated with the EZ DNA Methylation-Gold Kit (ZYMO, USA) according to the manufacturer's instructions. The modified DNA was amplified with the PCR primers described in Table S1. Methylation data are presented as the percentage of average methylation in all observed CpG sites. DNA methylation screen and pyrosequencing analysis were performed by SHBIO (Shanghai, China).

RNA in situ hybridization (ISH)

For the tissue microarray, the LINC00261 probe was labeled with digoxin (Exiqon, Denmark). The pancreatic tissue array was dewaxed and hydrated and then washed with PBS. The array was incubated with 15 μg/ml protease K at 37°C for 40 min. After washing with PBS, the array was dehydrated through a gradient of 70%, 96%, and 100% ethanol. Then, 50-100 μl of hybridization solution was added to each array. The array was covered on 22 × 22 glass and hybridized for 1 h at 50℃. Then, the array was washed with 5× SSC, 1× SSC, and 0.2× SSC at 50℃ and washed with PBS at room temperature. After washing the array was placed into the sealing solution and sealed for 15 min (1 ml sealing solution: 10× Roche sealing solution 100 μl/1× maleic acid buffer 900 μl). The sealant was dried with paper and incubated with probe fragments (1:800) overnight at 4°C. After incubation, the slide was washed with TBST. The reaction NBT/BCIP reaction was incubated for 1 h in a wet box in the dark (diluted NBT/BCIP buffer: 20 μl Roche reagent buffer/1 ml NBT/BCIP diluent). The slide was washed with TBST, and 200 μl of red nuclear fixation dye was added for 1 min. Then, the slide was placed under running water for 10 min and dehydrated with alcohol. The slide was sealed with glycerin buffer. The staining intensity score was defined as 0 (negative), 1 (weak), 2 (moderate), 3 (moderate-strong), and 4 (strong), and the positivity rate score was defined as follows: 0 (negative), 1 (1-25%), 2 (26-50%), 3 (51-75%), and 4 (76-100%). The product of the staining intensity score and positive rate score was considered the total ISH staining score. A total score of ≦ 4 was used for the low LINC00261 expression group, and a total score > 4 was used for the high LINC00261 expression group.

Fluorescence in situ hybridization (FISH)

For the PC cell lines, the LINC00261 probe was labeled with cyanine-3 (GenePharma, China). PC cells were fixed in formaldehyde, permeabilized with Triton X-100, and dehydrated with ethanol. The dried cells were mixed with 1 μg/μl of the FISH probe (sequence: see Table S1) in a hybridization buffer and incubated at 37 ℃ overnight. The following day, the slides were washed, dehydrated, and nuclei were finally stained using DAPI. Staining was performed, avoiding light, for 20 min, and cells were observed under a fluorescence microscope.

Immunohistochemistry (IHC) assay

c-Myc, Ki67 was detected in IHC. After de-waxing and hydration, antigen retrieval and blocking, the tissue microarray slide was incubated with specific primary antibodies overnight at 4 °C, and followed by the observation using IHC kit purchased from Zsbio (Beijing, China). Primary antibody of c-Myc was described in Table S1.

Western blot assay

Total protein was extracted from cell lysis prepared with cold, freshly RIPA buffer (Sigma, USA) and measured with a BCA Protein Assay kit (Beyotime, China) according to the manufacturer's protocol. The protein samples (30 μg) were separated by 4%-20% gradient SDS-polyacrylamide gel electrophoresis (PAGE, GenScript, USA) and transferred to polyvinylidene fluoride (PVDF, Millipore, USA) membranes for immunoblotting. The membranes were hybridized with a primary antibody at 4°C overnight with gentle shaking, and followed by incubation with a secondary antibody for 1 h at room temperature. Horseradish peroxidase-conjugated secondary antibodies (Cell Signaling Technology) and an ECL chemiluminescence kit (GE, USA) were used to detect bound antibodies. Information of used antibodies were as described in Table S1.

RNA extraction and quantitative real-time PCR (qRT-PCR)

Total RNA was extracted from tissues or cells using an Ultrapure RNA kit (Cwbio, China). The RNA concentration and purity were measured with NanoDrop ND-2000 spectrophotometer (Thermo, USA). Reverse transcription and quantitative real-time PCR were performed using a PrimeScript RT reagent kit and SYBR® Premix Ex Taq™ kit respectively (Takara, Japan) according to the manufacturer's protocol. Each test was repeated in triplicate. Relative mRNA expression levels were calculated using the 2-ΔΔCt method and normalized to GAPDH expression levels. All primer sequences used in the present study are shown in Table S1.

RNA immunoprecipitation

RIP was performed using a Magna RIP™ RNA-Binding Protein Immunoprecipitation kit (Merck Millipore, Germany) according to the manufacturer’s instructions. PANC-1 and SW1990 cells were lysed in complete RIP lysis buffer, and the cell extracts were incubated with magnetic beads conjugated to specific antibody or control IgG for 12 h at 4℃. Beads were washed and incubated with proteinase K to remove proteins. Finally, purified RNA was subjected to qRT-PCR analysis. The IgG antibody used in RIP (Merck Millipore, Germany).

Chromatin immunoprecipitation

ChIP experiments were performed using a MagnaChIP kit (Millipore) according to the manufacturer's instructions. For the ChIP experiment, PC cells were crosslinked with 1% formaldehyde, and the reaction was quenched by the addition of 125 mM glycine. Then, the cells were washed and lysed with cell lysis buffer, and chromatin was sheared to fragments of 100-500 bp by sonication for seven cycles at high amplitude (cycles of 15 s on followed by 45 s off). Then, 5 µg of specific antibody or IgG control antibody was adsorbed onto protein G magnetic beads and incubated with the chromatin extracts at 4°C overnight. Crosslinking of DNA fragments was reversed by ChIP elution buffer and subsequent incubation at 62°C for 2 h and 95°C for 15 min. Recovered DNA was subjected to RNase treatment and analyzed

via qPCR. Sequences of primers used for the ChIP-qPCR quantification are provided in Table S1.

Immunoprecipitation (IP)

After 48 hours of transfection, the PC cells were lysed in an immunoprecipitation lysate buffer (20 mM Tris-HCl, pH 8.0, 100 mM NaCl, 1% NP-40, and a protease inhibitor cocktail tablet) for 15 min on ice. The lysates were centrifuged at 10,000 g for 10 min at 4at 44 4or 10 min at 4min at 44 40 g for 10 mi collected into a new EP tube. The cell lysates were added to 2 μg primary antibody and incubated for 1 hour at 4°C. After incubation, 50 μl Protein A/G PLUS-Agarose (Santa Cruz, CA, USA) was added to the protein-antibody complexes and incubated at 4°C on a rotating device overnight. The immunoprecipitates were washed four times with immunoprecipitation buffer, and a 2x sample loading buffer was added to the beads before boiling for 5 min. The supernatant was collected and used in a Western blot assay.

Cell culture

The human PC cell lines BxPC-3, CFPAC-1, PANC-1 and SW1990 were purchased from American Type Culture Collection (ATCC, USA) and were incubated in complete growth medium with 10% fetal bovine serum (FBS, Gibco, USA), as recommended by the manufacturer. Cultured cells were maintained at 37°C in a humidified incubator with 5% CO2. All cell lines were fingerprinted for authenticity validation.

Cell Transfection and vectors construction

Knockdown siRNA for LINC00261, c-Myc and p300/CBP were purchased from RiboTM (Guangzhou, China). The overexpression plasmid of c-Myc , p300 and CBP was purchased from Genchem (Shanghai, China). Both the siRNAs and plasmids were transfected using Lipofectamine 3000 (Invitrogen, USA) following the manufacturer's protocol. Overexpression lentiviral vectors of LINC00261 were constructed by Hanbio Biotechnology (Shanghai, China). Lentiviral infection was performed according to the manufacturer's

instructions, and GFP or RFP-positive virus-infected cells were selected by puroc-Mycin. The mutant LINC00261 vectors were constructed by Sangong (Shanghai, China) and the mutant p300/CBP vectors were constructed by Genchem. The CRISPR/dCas9 system (EF1a-Dcas9-Tet1CD-CMV-EGFP/ sgLINC00261) was constructed by GeneChem. The system was used for reversing LINC00261 expression by demethylating the LINC00261 promoter.

Cell proliferation and viability assay

For the cell proliferation assay, a total of approximately 25000 PC cells were plated in 24-well plates. After 24 h of culture, EDU assay was performed to evaluate cell proliferation using a Cell Light™ EDU kit (RiboBio, Guangzhou, China) according to the manufacturer's instructions. Similarly, cell viability was assessed using a cell counting kit CCK8 (Dojinodo, China) according to the manufacturer's instructions, a total of approximately 3000 PC cells were plated in 96-well plates. All experiments were repeated in triplicate.

Cell cycle assay

For cell cycle analysis, cells were collected and fixed in 70% ethanol at 4°C overnight. Then the cells were incubated with Rnase and propidium iodide (Beyotime, China) according to the manufacturer's protocol. DNA content was measured using a BD Accuri C6 flow cytometer. All experiments were repeated independently in triplicate. The data were analyzed using the ModFit 3.3 software (BD Bioscience, Sparks, MD, USA).

Migration and invasion assay

For migration assays, 5×10^4 cells were suspended in 100 μl of serum-free DMEM and seeded in the top chamber of the transwell (8 μm, 24-well format, Millipore, USA). Then, complete medium with 10% FBS was added to the bottom chamber. For invasion assays, the transwell chambers were coated with 30 μl of basement membrane Matrigel (diluted 1:4 in DMEM, Corning Life Science, USA) for 5 h in a 37° incubator, and 1×10^5 cells were seeded in the top chamber. After 24h incubation, the chambers were fixed with 4%

paraformaldehyde for 30 min and stained with crystal violet (Beyotime, China). Photographs of three random fields were captured and the cells in the imaged fields were counted under a microscope. Three independent replicates of each experiment were performed.

In vivo animal experiments

Four- to six-week-old female athymic nude mice were purchased from Southwest Hospital (Chongqing, China). All mice were randomized to experimental or control group using a systematic random sampling according the serial number (such as: 1, 3, 5 in experimental group and 2, 4, 6 in control group). All of our animal experimental procedures were carried out in aseptic conditions. To establish the subcutaneously implanted tumor model, 2×10^6 cells (in a total volume of 0.1 ml of PBS) were injected into the dorsal region of each mouse. The body weight and tumor growth of each mouse were measured every week. All efforts were made to minimize suffering, and all mice were sacrificed for measurement of tumor weights 5 weeks after establishment of the model. To establish the metastasis model in nude mice, a midline incision was made in the anterior abdominal wall, and 2×10^6 cells (in a total volume of 0.1 ml of PBS) were directly injected into the distal pancreatic parenchyma. Mice were anesthetized with isoflurane inhalation or pentobarbital sodium. After 6 weeks, the liver metastatic ability of the PC cells was observed by harvesting of liver and pancreas tissues. No blinding method was done in the present study. The animal experiments were approved by the Institutional Animal Care and Use Committee of Southwest Hospital, Chongqing, China.

Statistical analysis

Continuous data were tested for normality using the Shapiro-Wilk test at first. Normally distributed continuous data of two groups were expressed as the mean ± standard deviation and compared using a t-test. Nonnormal continuous data or small sample data ($n<5$) of two groups were expressed as the median (interquartile range) and compared using the Wilcoxon test. Continuous and normal distributed data with three or more groups were tested for normality by evaluating the normality of the residuals using the Shapiro-Wilk test. Variance equality was then tested using Levene’s test. If the distribution was normal and variance is

equal, data were compared using ANOVA and post-Hoc analyses were performed using Turkey's test. If the distribution was normal and variation is unequal, the ANOVA was corrected using the Welch method and multiple comparisons were performed using the Games-howell's test. If the distribution is nonnormal, Kruskal Wallis test was used as appropriate. Categorical data were expressed as frequencies (percentages) and compared using the Chi-square test. Kaplan-Meier curve, univariate and multivariate Cox regression were performed to analyze survival data. Final screens of covariates and selection of the best-fitted Cox regression model were performed using a stepwise method. Nomogram was developed based on the independent predictors identified by multivariate Cox analysis. For the validation of nomogram, discrimination was evaluated using the C-index (area under curve, AUC) and calibration was evaluated by analyzing observed and expected outcome events followed with Hosmer and Lemeshow Test. $P > 0.05$ indicated a good performance of discrimination. For in vitro, all experiments were biologically repeated for three times according to the routines of previous studies. All error bar represented standard error of mean. All statistical analyses were performed using R (version 3.6.1, https://www.r-project.org/). All tests were two-sided, and $P<0.05$ was considered statistically significant and expressed as *.

Consent form (Patient section, censored version)

I authorize my physician to dispose my diseased organ, tissue or specimen for proper purposes, including pathological test, cytological test, medical waste treatment or other use.

Patient signature: ____________________

Patient's family member or proxy: ________Relationship with the patient: _____________

Reasons why patients cannot sign: ____

Date: Year___________Month_____________Day____________

Table S3

Baseline Characteristics of the Study Participants Stratified by LINC00261 Expression Levels

Variables	LINC00261 expression levels		
	Low (n=103)	High (n=102)	*P*
Age, years, mean ± SD	61.46 ± 10.88	61.14 ± 11.23	0.837
Gender, n (%)			0.818
Female	41 (39.8)	39 (38.2)	
Male	62 (60.2)	63 (61.8)	
Local invasion, n (%)			0.203
No	77 (74.8)	68 (66.7)	
Yes	26 (25.2)	34 (33.3)	
Peri-nerual invasion, n (%)			0.702
No	64 (62.1)	66 (64.7)	
Yes	39 (37.9)	36 (35.3)	
Lymphnode metastasis, n (%)			<0.001
No	52 (50.5)	78 (76.5)	
Yes	51 (49.5)	24 (23.5)	
Tumor size, cm^3, M (IQR)	23.00 (39.00)	17.75 (26.53)	0.030
Differentiation, n (%)			0.018
Well	14 (13.6)	14 (13.7)	
Medium	61 (59.2)	76 (74.5)	
Poor	28 (27.2)	12 (11.8)	
Clinical stage, n (%)			<0.001
Ia-IIa	44 (42.7)	71 (69.6)	
IIb-IV	59 (57.3)	31 (30.4)	

SD denotes standard deviation; M (IQR) denotes median (inter-quartile range)

Table S4

Univariate and Multivariate Cox Proportional Hazard Regression on the Association between LINC00261 Expression Levels and Survival

	Univariate		Multivariate[a]	
Characteristics	HR (95%CI)	*P*	HR (95%CI)	*P*
Age, years, continuous	1.02 (1.00-1.04)	0.026	1.03 (1.01-1.05)	0.001
Gender, male vs female	1.18 (0.83-1.67)	0.361	-	-
Local invasion, yes vs no	1.14 (0.79-1.64)	0.486	-	-
Peri-neural invasion, yes vs no	1.06 (0.75-1.51)	0.729	-	-
Lymphnode metastasis, yes vs no	1.94 (1.38-2.74)	<0.001	-	-
Tumor size, >21cm vs ≤ 21cm	1.22 (0.87-1.72)	0.257	-	-
Differentiation, medium vs well	2.56 (1.19-5.52)	0.016	2.31 (1.06-5.01)	0.034
Differentiation, poor vs well	4.13 (1.82-9.36)	<0.001	3.24 (1.40-7.48)	0.006
Clinical stage, IIb-IV vs I-IIa	2.06 (1.46-2.90)	<0.001	1.85 (1.28-2.67)	0.001
LINC00261, high vs low	0.47 (0.33-0.67)	<0.001	0.60 (0.42-0.87)	0.006

HR (95%CI) denotes hazard ratio (95% confidence interval); [a]Best fit of multivariate model is determined using a stepwise method base on the AIC (Akaike Information Criterion) statistic.
